# The conversion of evodiamine-induced hepatotoxicity into a therapeutic effect on colonitis: insight from the liver-gut axis mediated by PPAR/NF-κB/ZO-1/caspase-3 pathway

**DOI:** 10.1186/s13020-025-01262-3

**Published:** 2025-11-21

**Authors:** Chongjun Zhao, Qiqi Fan, Ying Dong, Shuang Sun, Yao Zhang, Haiqiang Yao, Hongming Ren, Jiaqi Li, Chuanqi Qiao, Jian Li, Gaimei She, Ruichao Lin

**Affiliations:** 1 Beijing Key Laboratory for Quality Evaluation of Chinese Materia Medica, Beijing University of Chinese Medicine, Beijing, 100102 China; 2 Traditional Chinese Medicine Processing Technology Inheritance Base of National Administration of Traditional Chinese Medicine, Beijing University of Chinese Medicine, Beijing, 100102 China; 3 Xiyuan Hospital, China Academy of Chinese Medical Sciences, Beijing, 100091 China; 4 School of Traditional Chinese Medicine, Beijing University of Chinese Medicine, Beijing, 100029 China

**Keywords:** Evodiamine, Hepatotoxicity, Inflammatory bowel disease, Gut-liver axis

## Abstract

**Background:**

Evodiamine (EVO) exerts promising therapeutic potential in the treatment of Ulcerative Colitis (UC). However, its clinical application is constrained by concerns regarding potential hepatotoxicity. A comprehensive understanding of underlying both the therapeutic effects and hepatotoxicity of EVO is therefore essential to enhance its safe and effective application in clinical practice.

**Purpose:**

This study aimed to elucidate the regulatory mechanisms of gut-liver axis homeostasis in EVO-induced hepatotoxicity and its therapeutic effects on UC.

**Methods:**

An integrated experimental strategy employing cell, zebrafish, and murine was implemented to assess the hepatotoxic effects of EVO. Transcriptomic and metabolomic analyses were performed in vitro, while targeted investigations of bile acids (BAs) metabolism were conducted in vivo to understand the overall response profile and the underlying mechanisms associated with EVO-induced hepatotoxicity. Furthermore, the expression patterns of proteins along the gut-liver axis were systematically evaluated under diverse physiological conditions to identify the potential interactions contributing to the alleviative effects of UC on EVO-induced hepatotoxicity and as well as to explore the therapeutic potential of EVO in UC management.

**Results:**

High-dose EVO treatment was associated with notable hepatotoxic effects in both in vitro cellular models and normal in vivo animals, primarily manifested through disturbances in BAs metabolism, inflammatory responses, and apoptosis. In contrast, in UC models, EVO administration not only effectively ameliorated intestinal structural damage and functional impairments, but also demonstrated minimal hepatotoxicity. Mechanism studies documented that EVO disrupted bile acid metabolism by interfering with BSEP/MRP2/CYP7A1/CYP27A1 pathways, while simultaneously triggering inflammation and apoptosis through PPAR/NF-κB/ZO-1/caspase-3 pathway, ultimately contributing to hepatotoxicity in healthy subjects. However, in the context of UC, the disease condition attenuated EVO-induced alterations in hepatic protein expression, thereby reducing its hepatotoxic potential. Meanwhile, under UC conditions, EVO restored the expression levels of relevant proteins in the intestinal tract, thereby maintaining its therapeutic efficacy against UC.

**Conclusion:**

The hepatotoxicity observed under healthy conditions and the therapeutic efficacy of EVO against UC are both associated with EVO's modulation of the PPAR/NF-κB/ZO-1/caspase-3 pathway. The influence of EVO on the expression of these key proteins within the gut-liver axis may be modulated by distinct physiological states, resulting in either antagonistic or synergistic effects that potentially lead to differential biological responses across multiple organs. This study not only provides essential supplementation and refinement to the understanding of EVO-induced hepatotoxicity but also identifies a novel breakthrough in accurately assessing its liver toxicity. Specifically, the evaluation of EVO's hepatotoxic potential should be grounded in the principles of TCM and aligned with its clinical application characteristics. Furthermore, these findings offer valuable insights for the safety assessment and development of traditional Chinese medicines with potential hepatotoxic risks.

**Graphical Abstract:**

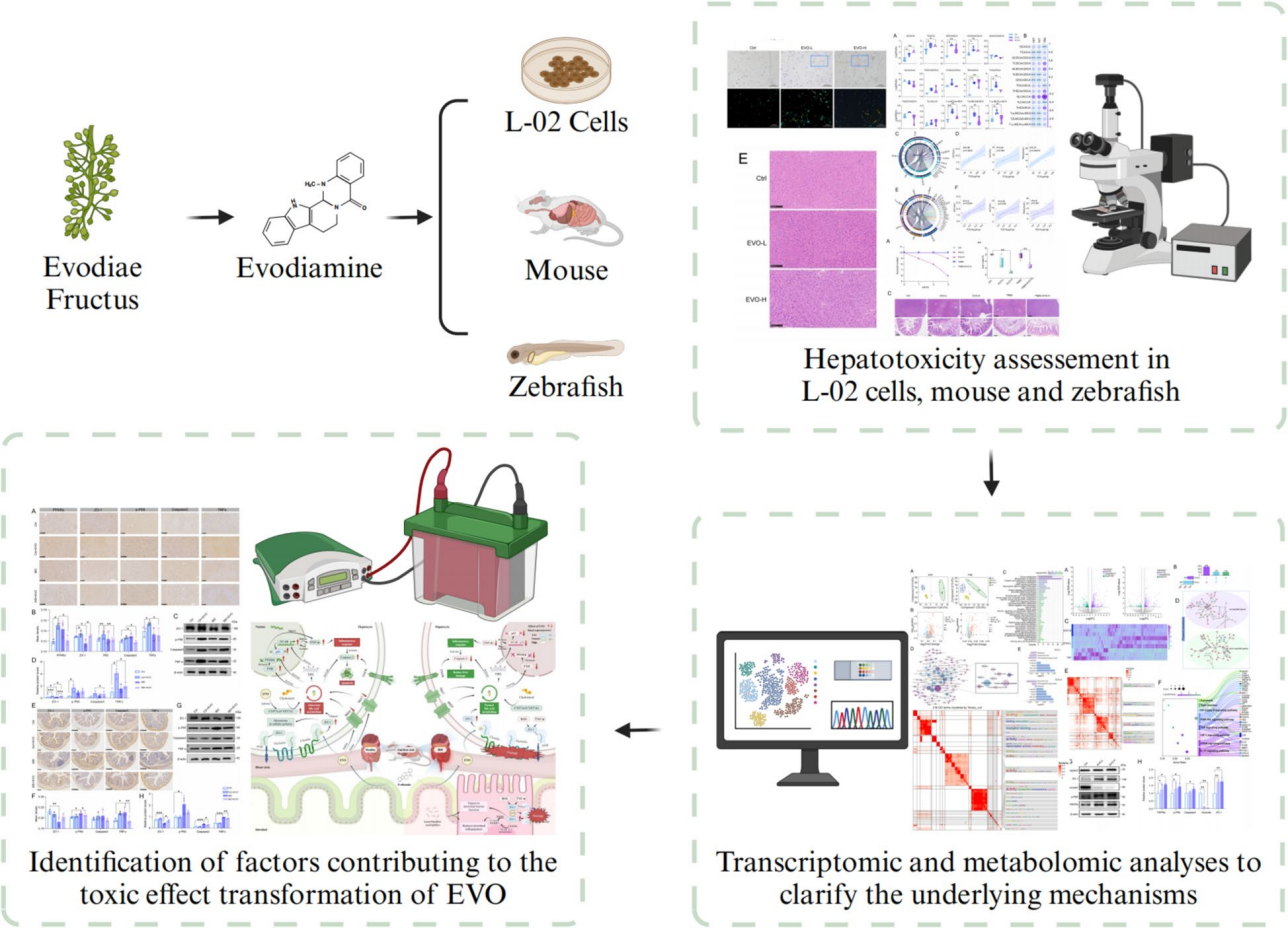

**Supplementary Information:**

The online version contains supplementary material available at 10.1186/s13020-025-01262-3.

## Introduction

Inflammatory Bowel Disease (IBD) is a chronic and increasingly prevalent global health condition, primarily encompassing Ulcerative Colitis (UC) and Crohn’s Disease (CD) [1]. The pathogenesis of IBD is widely regarded as multifactorial, involving a complex interplay of genetic predispositions, immune system dysregulation, and disruptions in the composition and function of the gut microbiota [2]. Despite significant advances in medical research, IBD remains a lifelong condition without a definitive cure, with current therapeutic approaches largely confined to surgical procedures or pharmacological interventions designed to mitigate symptoms and modulate disease progression.

Evodiamine (EVO), an alkaloid derived from Evodiae Fructus, features a complex multi-ring architecture anchored by a favorable indole scaffold. This intricate structure endows EVO with extended pharmacological properties, including anti-inflammatory [3], analgesic [4], anti-tumor [5], nervous system protective activity [6], and so forth. Of particular significance is the accumulating evidence underscoring EVO's remarkable therapeutic efficacy in the management of gastrointestinal disorders [7]. Notably, studies have demonstrated that EVO exerts protective effects against UC by enhancing the abundance of Lactobacillus acidophilus and promoting the production of beneficial intestinal acetate [8, 9]. Furthermore, EVO has been shown to suppress NLRP3 inflammasome activation through autophagosome-mediated degradation and to modulate the NF-κB signaling pathway [10]. These results collectively position EVO as a compelling candidate with immense therapeutic promise for future biomedical applications.

Drug safety occupies a position of utmost significance within clinical practice [11]. Numerous pharmaceutical agents with remarkable pharmacological efficacy are frequently accompanied by undesirable side effects [12, 13], which not only curtail their therapeutic applicability but also heighten the potential risks inherent in clinical administration [14]. Currently, EVO finds itself in a predicament wherein its otherwise promising clinical prospects are hindered by documented potential hepatotoxicity [15], cardiotoxicity [16] and nephrotoxicity [17]. Although prior investigations have provided valuable insights into the toxicological profile of EVO, these studies have employed diverse experimental models, dosage regimens, and evaluation criteria, resulting in an inconsistent and fragmented understanding of its organ-specific toxic effects. Recently, we undertook a comprehensive assessment of EVO's toxicological profile in zebrafish, revealing that hepatotoxicity manifests earlier than cardiotoxicity. Moreover, the dosage of EVO necessary to provoke nephrotoxicity in murine is markedly higher than that employed to evaluate hepatotoxicity [4]. Nevertheless, the molecular underpinnings of EVO-associated toxicity remain incompletely understood. Therefore, it is of importance to develop a rigorous and systematic framework to extract meaningful insights from the complex toxic manifestations of EVO and to delineate the mechanisms involved.

Recently, extensive evidence has illuminated the intricate bidirectional communication within the gut-liver axis, highlighting its pivotal role in the initiation, progression, and modulation of various diseases, as well as in determining drug responses and toxicity profiles [18–21]. Furthermore, therapeutic strategies that adopt a holistic, gut-liver axis-centered perspective–are increasingly being recognized for their potential in managing both gastrointestinal and hepatic disorders. As previously outlined, EVO demonstrates multi-effects on this axis-some of which appear contradictory–necessitating further comprehensive investigation to decipher the delicate balance between its therapeutic potential and adverse impacts, which resonates with the integrative principles of traditional Chinese medicine (TCM) and aligns with the TCM-guided philosophy of "precise medication" and toxicity regulation.

In this study, we systematically investigated the hepatotoxic effects of EVO through both in vitro cellular models and in vivo experimental systems. To achieve comprehensive understanding of the underlying mechanisms, integrated multi-omics analyses were performed and subsequently validated using RT-PCR and western blot. Additionally, we evaluated the therapeutic potential of EVO in the context of UC and compared its hepatotoxic profile under healthy versus UC conditions. Our findings revealed that EVO-induced hepatotoxicity was associated with disturbances in bile acid (BA) and lipid metabolism, as well as inflammatory responses, which were regulated by alterated expression of key proteins including PPAR, NF-κB, TJs, BSEP, MRP2, CYP7A1, and CYP27A1. More importantly, EVO-induced hepatotoxicity was alleviated in UC mice compared to healthy controls, while concurrently exerting a protective effect against intestinal injury. These observations may be attributed to distinct alterations in protein expression along the gut-liver axis resulting from the EVO and UC. Therefore, the intertwined and inseparable pharmacological effects of EVO in treating UC and hepatotoxicity may be closely linked to the diverse states of gut-liver axis, ranging from healthy conditions to those associated with UC. This underscores the importance of appropriate application of potentially toxic TCM, which is essential not only for effective disease management but also for ensuring comprehensive clinical safety, including the mitigation and complete transformation of toxic effects.

## Materials and methods

### Evaluation of hepatotoxicity in vitro cell

#### Cell incubation and viability assay

The normal human liver cell line (L02 cells), obtained from Cell Resource Center, IBMS, CAMS/PUMC (Beijing, China) were cultured in complete RPMI-1640 medium (Procell Life Science & Technology Co., Ltd., Wuhan, China) supplemented with 10% FBS, 100  U/mL of penicillin and 100 µg/mL of streptomycin (CORNING Co. Ltd., NY, USA) at 37 ℃ with 5% CO_2_. Cells were seeded in 96-well plates at a density of 5 × 103 cells/well for 24 h. The cells were treated with EVO (200,412, Shanghai Yuanye Bio-Technology Co., Ltd., Shanghai, China) at various concentrations for 24 h or 48 h. CCK8 solution (Beijing Lablead Biotechnology Co., Ltd., Beijing, China) was added to each well, followed by incubation at 37℃ for 2 h. The OD values were measured at 450 nm on a microplate reader (BioTek Instruments, Inc., VT, USA).

### Biochemical indexes

L02 cells were seeded in 6-well plates at a density of 1.5 × 105 cells/well and incubated for 24 h. Subsequently, the cells were treated with either complete medium alone, 0.15 μg/mL of EVO (EVO-L) or 0.3 μg/mL of EVO (EVO-H) for an additional 24 h. Thereafter, both cell samples and the supernatant were collected and subjected to biochemical evaluation using Alanine transaminase (ALT), aspartate aminotransferase (AST), total bile acid (TBA), total cholesterol (TC) and triglyceride (TG) assay kits (Nanjing Jiancheng Bioengineering Institute, Nanjing, China).

### Cell apoptosis staining and flow cytometry analysis

The cells were processed in accordance with the procedures outlined in the "Biochemical indexes" section. Specifically, cells were digested using an EDTA-free trypsin solution, washed with PBS, and centrifuged at 1000 rpm for 5 min. Subsequently, the cells were resuspended in 1X binding buffer to achieve a concentration of 1 × 10^6^ cells/mL. Apoptosis detection was performed using the Annexin V-FITC/PI Apoptosis Detection Kit (C1002, Beijing Lablead Biotechnology Co., Ltd., Beijing, China) according to the manufacturer's instructions. The apoptotic rate was determined using a BD FACSCelesta™ Cell Analyzer (Becton, Dickinson and Company, NJ, USA), and the resulting data were analyzed using FlowJo software (version 10).

### Untargeted metabolomic analysis

The 1.5 × 10^7^ cells from different groups were harvested and extracted using 400 µL acetonitrile: methanol (1:1, v/v). Subsequently, the samples were centrifuged at 13,000 g for 15 min at 4 °C. The resulting supernatant was dried with nitrogen gas, followed by reconstitution in 120 µL of acetonitrile: water (1:1, v/v) solution. After a second centrifugation step under the same conditions, the final supernatant was transferred to autosampler vials for metabolomics analysis. The quality control (QC) samples were prepared by mixing equal volumes of the analytic samples. The metabolite separation was performed on an ExionLC AD system coupled with a Triple TOF5600 analyzer (AB Sciex Pte. Ltd., MA, USA) equipped with an ACQUITY BEH C18 column (100 mm × 2.1 mm i.d, 1.7 µm; Waters Corporation, MA, USA). The mobile phases A and B consisted of 0.1% formic acid in water and a mixture of acetonitrile/isopropanol (1:1) containing 0.1% formic acid, respectively. The elution gradient was programmed as follows: 0–3 min, 95%–80% A; 3–9 min, 80%-5% A; 9–13 min, 5% A; 13.0–13.1 min, 5%-95% A; 13.1–16 min, 95% A. Each injection volume was 10 μL, with a flow rate of 0.40 mL/min while maintaining a column temperature at 40 °C. For the mass spectrometry conditions, both positive and negative ion scanning modes were employed within a mass scanning range between m/z 50–1000. The spray voltages for positive and negative ions were 5000 V and 4000 V, respectively. The declustering potential was maintained at a constant value of 80V. The nebulizer gas, auxiliary heating gas, and curtain gas were established at 50 psi, 50 psi, and 30 psi, respectively. The ion source heating temperature was maintained at 500 °C, and the collision energy was cycled between 20 and 60 V. The raw data were imported into the Progenesis QI 2.3 (Nonlinear Dynamics, Waters Corporation, MA, USA) for peak detection and alignment. The metabolites with statistically significant difference were selected with VIP ≥ 1 and p ≤ 0.05, and subsequently subjected to metabolic enrichment and pathway analysis (http://www.genome.jp/kegg/).

### Transcriptomic analysis

Total RNA was extracted using Trizol reagent (Illumina, Inc., CA, USA). The RNA concentrations were quantified by NanoDrop2000 (Thermo Fisher, MA, USA), and the integrity was evaluated on 2100 Bioanalyzer with the RNA 6000 Nano Kit (Agilent Technologies, CA, USA). Libraries were constructed by the TruSeq Stranded mRNA Kit (Illumina, Inc., CA, USA). Specifically, polyA-tailed RNA molecules were enriched using Oligo magnetic beads and fragmented with fragmentation buffer. Subsequently, double-stranded cDNA synthesis was carried out. Following adenylation of the 3'ends and cDNA amplification, target fragments were recovered for quantization on a 2% agarose gel. The final libraries were subjected to ultra-high throughput sequencing based on Illumina Novaseq 6000 (Illumina, CA, USA). The clean reads were aligned to the human genome using HISAT2 (http://ccb.jhu.edu/software/hisat2/index.shtml), and differential analysis of gene expression was conducted using edgeR (http://www.bioconductor.org), followed by multiple testing correction by the Benjamini–Hochberg procedure.

### Evaluation of hepatotoxicity in animals

#### Evaluation of hepatotoxicity in mice

Male C57BL/6J mice (18–20 g) were supplied by SPF (Beijing) Biotechnology Co. Ltd. (SCXK (Jing) 2019–0010) and accommodated in a specific-pathogen-free (SPF) environment with unrestricted access to food and water. Following a 1-week acclimatization period, all animal experiments were performed in accordance with the guidelines established by the National Research Council and approved by the Animal Ethical Committee of Beijing University of Chinese Medicine (BUCM-2024040803–2015). The mice were randomly assigned into 3 groups (n = 6 per group), namely the control group (Ctrl), EVO treatment groups (800mg/kg/d for EVO-L and 1600 mg/kg/d for EVO-H, orally on a daily basis). Mice in the Ctrl group were orally administered daily with 0.5%CMC-Na. After 7 days of administration, the mice were euthanized and the serum and liver were collected for subsequent experiments.

### The hepatotoxicity comparison of EVO in UC and healthy mice

Four experimental groups were established, namely the Ctrl, the mice treated with EVO group (Ctrl + EVO), the dextran sulfate sodium (DSS; Dalian Meilun Biotechnology Co., Ltd., Liaoning, China) -induced UC group (UC), and the EVO-treated UC group (UC + EVO). Mice in UC and UC + EVO groups were administered with 3% (w/v) DSS via free access to drinking water for 7 consecutive days. In contrast, mice in Ctrl and Ctrl + EVO groups received regular water intake. Following the successful induction of UC, mice in Ctrl + EVO and UC + EVO groups were administered 800 mg/kg/d of EVO orally on a daily basis, whereas the other groups of mice received an equivalent volume vehicle. The body weight of all mice was monitored daily throughout the experimental period. At the end of the treatment period, the mice were euthanized and the liver, colon and serum were collected for further analysis.

### Targeted metabolism of BAs

25 mg of liver tissue from different groups was accurately weighed and subjected to metabolism extraction. A volume of 350 μL extraction solvent (methanol: water = 4:1) was added, followed by homogenization using a cryogenic grinding instrument at − 10°C with a frequency of 50 Hz for 6 min. The resulting mixture was then subjected to low-temperature ultrasonication at 5 °C and 40 kHz for 30 min. After incubation at – 20 °C for 30 min, the sample was centrifuged at 13,000 rcf at 4 °C for 15 min. The supernatant was collected, evaporated to dryness under a gentle stream of nitrogen, and reconstituted in 100 μL of 50% acetonitrile in water. The reconstituted samples were subjected to a second round of low-temperature ultrasonication and centrifugation under the same conditions, and the final supernatant was collected for instrumental analysis. An ExionLC AD system equipped with a BEH C18 column (150mm × 2.1mm, 1.7 μm; Waters, USA) was employed for chromatographic separation. The column temperature was controlled at 40°C and the injection volume was set to 5 μL. The mobile phases consisted of A (0.1% formic acid in water) and B (0.1% formic acid in acetonitrile). Mass spectrometry detection was performed using AB SCIEX QTRAP 6500 + instrument (AB Sciex Pte. Ltd., USA) in negative ion mode. The Curtain Gas was adjusted to 35 arbitrary units, the Collision Gas was set to Medium, and the IonSpray Voltage was configured to – 4500 V. The Temperature of the ion source was maintained at 550 °C, while the Ion Source Gas1 and Ion Source Gas2 were both set to 50 °C.

### Evaluation of hepatotoxicity in health and UC zebrafish

Mature zebrafish (AB strain) were procured from the China Zebrafish Resource Center (Wuhan, China). The zebrafish were maintained in a centralized aquaculture system under controlled conditions: a water temperature of 28.5 °C, a photoperiod of 14 h of light and 10 h of darkness, and feeding with artemia three times daily. Healthy, mature zebrafish aged 5 months were randomly allocated into 5 groups (n = 10 per group), namely the Ctrl, the 0.8 mg/L EVO (EVO-L) group, the 1.6 mg/L EVO (EVO-H), the TNBS-induced UC group (TNBS), and the TNBS + EVO-H group. Zebrafish in the TNBS and TNBS + EVO-H groups were exposed to 3.3 mg/L TNBS, while those in the Ctrl group were exposed to 0.1% DMSO. After 3 days of exposure, zebrafish livers and intestinal tissues were collected and fixed in 4% paraformaldehyde solution for HE staining.

### Quantitative real-time PCR (RT-qPCR)

Total RNA was extracted from liver samples with the RNA Easy Fast Tissue/Cell Kit (TIANGEN BIOTECH Co., Ltd., Beijing, China). The concentration and purity of the isolated RNA were determined via a NanoDrop 2000 spectrophotometer (Thermo Fisher, MA, USA). Reverse transcription was accomplished by the All-in-One First-Strand Synthesis Kit (Beijing Lablead Biotechnology Co., Ltd., Beijing, China). The resulting cDNA was utilized as the template for quantitative real-time PCR (qPCR) amplification, which was accomplished according to the instructions of the 2 × Realab Green PCR Fast mixture kit (Beijing Lablead Biotechnology Co., Ltd., Beijing, China) on a StepOne Plus RT-PCR System (Thermo Fisher, MA, USA). The primer sequences were listed in Supplementary Table 1.

### Western blot

Cells and liver tissues were homogenized and lysed in RIPA lysis buffer (Beyotime Biotech Inc., Shanghai, China) supplemented with protease and phosphatase inhibitor cocktail (NCM Biotech, Jiangsu, China). The protein concentrations were standardized across samples, after which 5 × SDS-PAGE Loading Buffer (NCM Biotech, Jiangsu, China) was added, and proteins were denatured by heating at 100 °C for 15 min. Subsequently, the proteins were separated via SDS-PAGE using 5% stacking gels at 60 V and 12% resolving gels (Beijing Aoqing Biotechnology Co., Ltd., Beijing, China) at 100 V, followed by transfer to PVDF membranes. Membranes were blocked with 5% nonfat-dried milk (BioRuler, CT, USA) and incubated overnight at 4 °C with the corresponding primary antibodies. Following incubation with HRP-conjugated secondary antibodies, protein bands were visualized with ECL substrate (NCM Biotech, Jiangsu, China) and detected in the ImageQuant LAS 4000 imager (General Electric Company, MA, USA). Protein levels were semi-quantified by ImageJ software (National Institutes of Health, MD, USA), and normalized to β-actin expression. Detailed information for antibodies is listed in Supplementary Table 2.

### Statistical analysis

Data are shown as mean ± standard deviation (SD), as indicated in the figure legends. The statistical difference between the two groups was determined using Student's t-test unless otherwise mentioned. For comparisons involving more than two groups, a one-way analysis of variance (ANOVA) was employed.

## Results

### EVO induces significant cellular apoptosis in cell

**Fig. 1:**
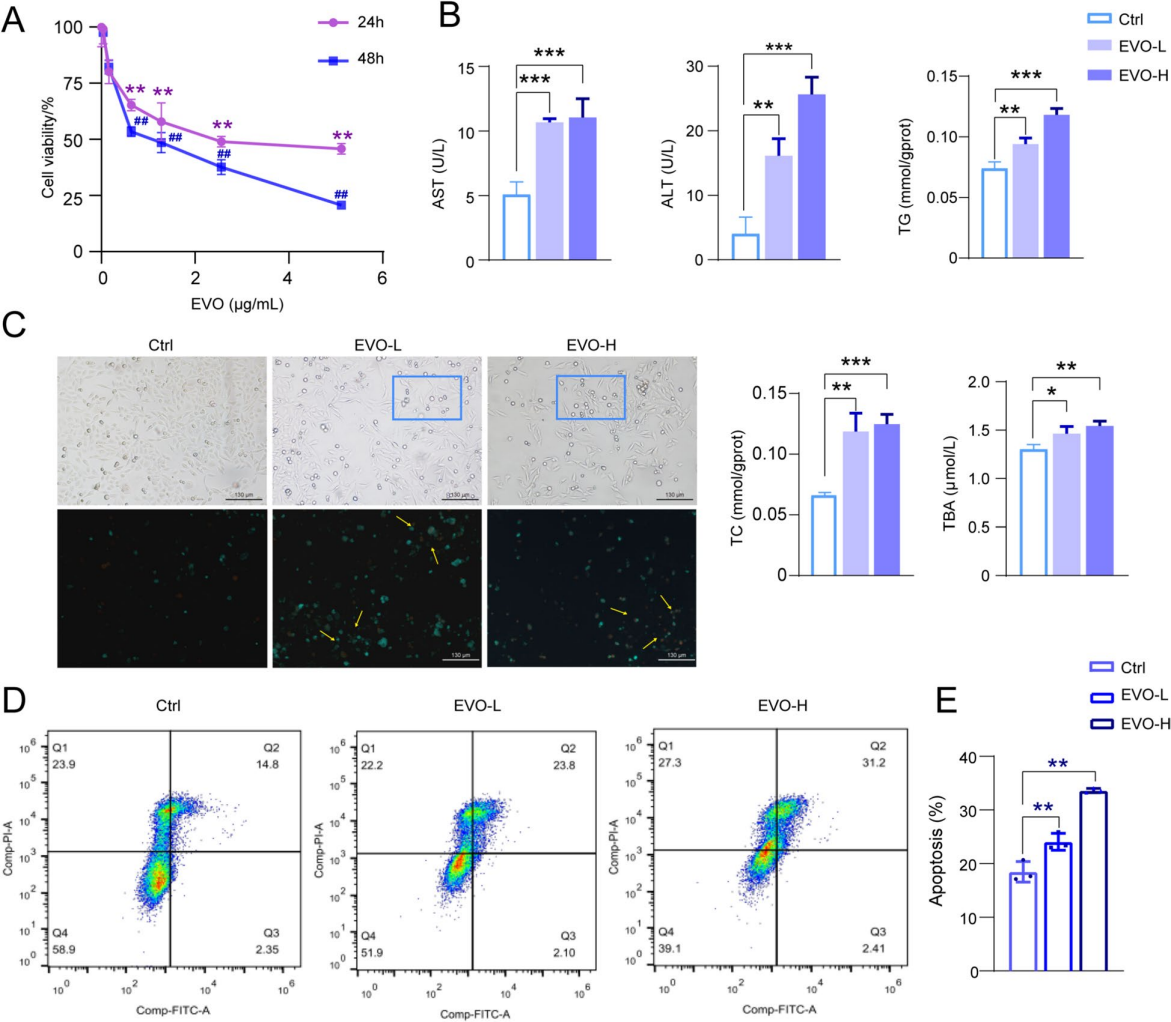
Effects of EVO on L02 Cells. **A** Cell viability. **B** Evaluation of ALT, AST, TC, TG and TBA. **C** The apoptotic status of L02 cells observed under the microscope. **D**, **E** The effect of EVO on L02 cells apoptosis. *P < 0.05; **P < 0.01; ***P < 0.001

To comprehensively characterize the toxicological profile of EVO, cell viability following 24-h and 48-h exposures to EVO was assessed using the CCK-8 assay. As illustrated in Fig. 1A, a marked and dose-dependent reduction in cell viability was observed commencing at a concentration of 160 ng/mL (*p* < 0.01). Compared with the Ctrl group, the EVO-L group (160 ng/mL) exhibited a significant elevation in ALT activity (*p* < 0.05), whereas the EVO-H group (180 ng/mL) displayed a significant increase in both ALT and AST levels (*p* < 0.05). Furthermore, notable increases in TC, TG, and TBA levels were detected in both the EVO-L and EVO-H groups (*p* < 0.05) (Fig. 1B). Taken together, these results collectively indicate that EVO induces cytotoxic effects on L02 cells and exerts detrimental influences on transaminase activity, lipid metabolism, and BAs homeostasis. Morphological assessment revealed that control cells maintained uniform cell density and predominantly spindle-shaped morphology with only a minimal presence of apoptotic bodies. In contrast, the EVO-treated group exhibited markedly inhibited proliferation, reduced cellular density, and elongated, wrinkled morphologies with branching structures. Notably, a substantial increase in apoptotic bodies was observed in the EVO-treated group (Fig. 1C), which was consistent with the result obtained from Annexin V and PI staining (*p* < 0.01) (Fig. 1D and E). Collectively, these results strongly support the conclusion that EVO effectively induces apoptosis in cells.

### EVO disrupted the synthesis and secretion pathways of BAs in cells

**Fig. 2:**
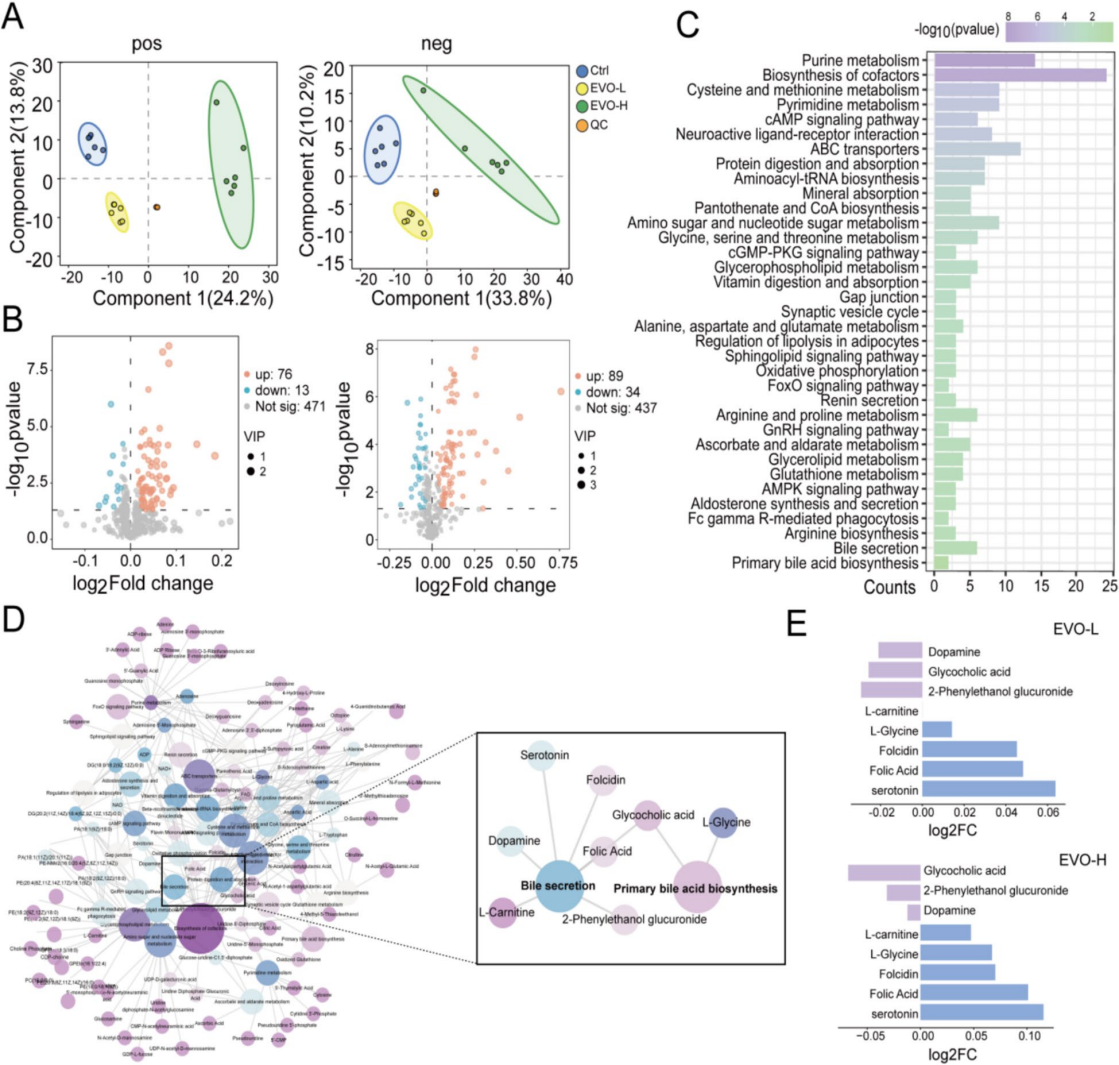
The effect of EVO on the metabolome of L02 cells. **A** PLS-DA score plots. **B** Volcanic diagram of metabolites. **C** Top 35 enriched KEGG pathway. **D** The interaction network between metabolites and metabolic pathways. **E** The abundance of dopamine, glycocholic acid, and 2-phenylethanol glucuronide in each group

Metabolomics was employed to elucidate the endogenous metabolic profiles modulated by EVO in cellular systems. Principal Component Analysis (PCA) and Partial Least Squares Discriminant Analysis (PLS-DA) results (Fig. 2A and Supplementary Fig. 1) revealed a high degree of clustering among QC samples, affirming the stability and reliability of the metabolomics methodology. Pronounced metabolic differences were observed between the Ctrl and both the EVO-L and EVO-H groups based on OPLS-DA models (Supplementary Fig. 2). Furthermore, the model's explanatory and predictive power was rigorously validated, with results indicating robustness, reliability, and high predictive accuracy (Supplementary Table 3). According to the criteria of VIP ≥ 1 and p ≤ 0.05, 89 differential metabolites were identified in the EVO-L group, whereas 123 in the EVO-H group (Fig. 2B). Through comprehensive and integrative analysis of both treatment groups, 54 differential metabolites were ultimately identified, predominantly characterized as endogenous metabolites potentially linked to the EVO-induced hepatotoxicity. KEGG enrichment analysis further revealed that these metabolites were significantly involved in several biological pathways, including purine metabolism, cysteine and methionine metabolism, pyrimidine metabolism, and the cAMP signaling pathway (Fig. 2C). Notably, pathways related to bile acid (BA) secretion and primary BA biosynthesis exhibited particularly significant enrichment. As depicted in the network diagram (Fig. 2D), a complex and highly interconnected interaction network was observed among these endogenous metabolites and their associated metabolic pathways. Moreover, consistent alterations in key metabolites such as dopamine, glycocholic acid, and 2-phenylethanol glucuronide were detected across the EVO-treated groups, specifically within the bile secretion and primary BA biosynthesis pathways (Fig. 2E). Collectively, these findings offer compelling evidence supporting the potential influence of EVO on BA synthesis and secretion in cells.

### EVO-induced hepatotoxicity is associated with PPAR/NF-κB/TJ/apoptosis pathway in cell

**Fig. 3:**
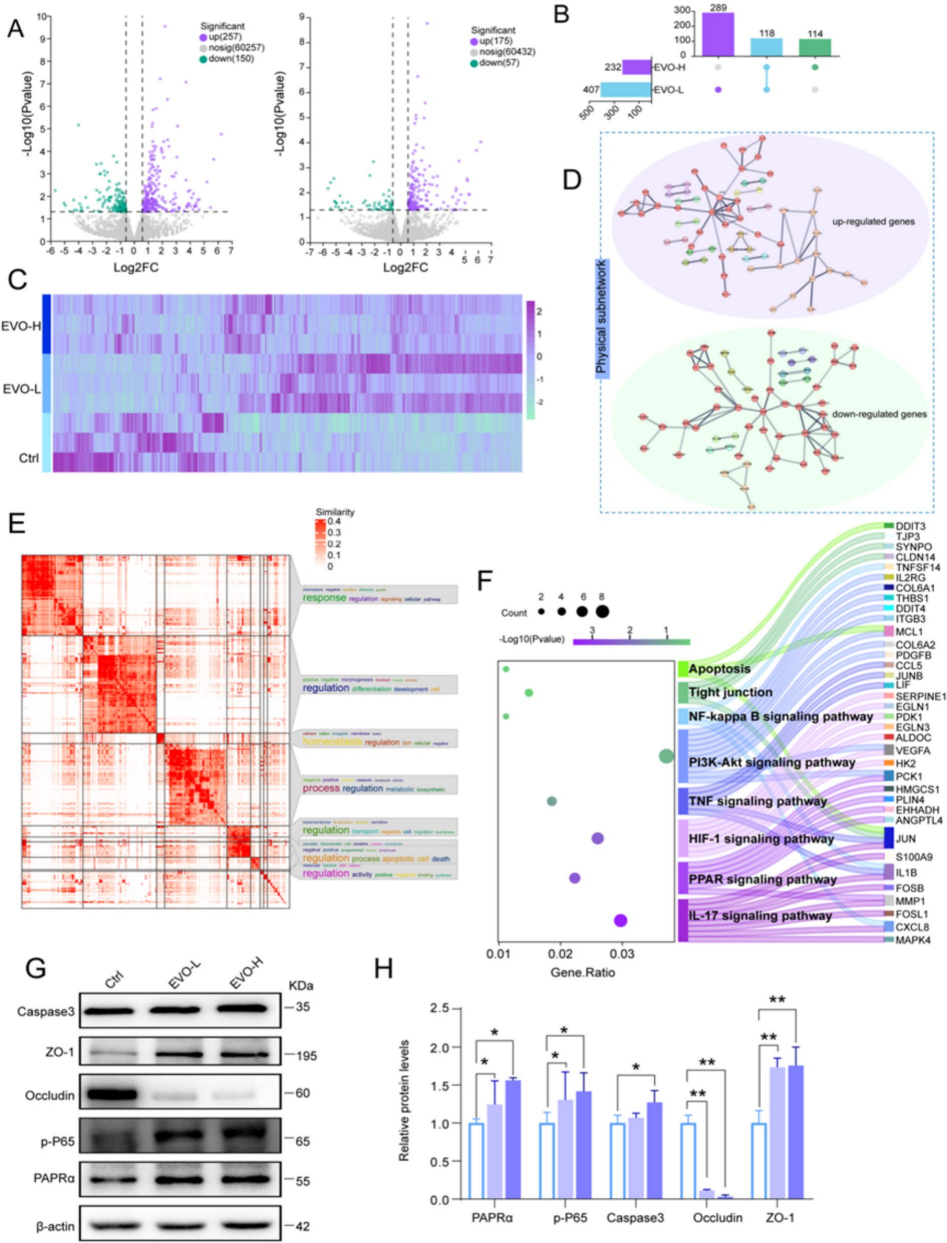
The impact of EVO on the transcriptome of L02 cells. **A** Volcanic diagram of DEGs. **B** The plot of common DEGs shared between the EVO-L and EVO-H groups. **C** The heatmap for the relative expression levels of DEGs across the three groups. **D** The physical subnetwork among these DEGs. **E** The items of BP category from GO annotation. **F** The important KEGG pathways along with their associated DEGs. **G**–**H** The expression levels of PPARα, p-P65, Occludin, ZO-1, Caspase3 proteins in L02 cells treated with EVO. *P < 0.05; **P < 0.01; ***P < 0.001

To explore the molecular mechanisms underlying EVO-induced hepatotoxicity, transcriptomic analysis was employed to characterize the global response patterns of L02 cells. Differentially expressed genes (DEGs) were meticulously rigorously identified and represented in a volcano plot (Fig. 3A). Compared with the Ctrl, the EVO-L group exhibited 407 significantly altered DEGs, among which 257 were upregulated. In the EVO-H group, a total of 232 DEGs were detected, including 175 upregulated genes. Integrated analysis of DEGs confirmed 521 potential targets associated with the cytotoxic effects of EVO on L02 cells (Fig. 3B) and the expression profiles of these DEGs across groups were summarized (Fig. 3C). Moreover, the principal physical subnetwork among these DEGs revealed a complex web of regulatory interactions and functional dependencies (Fig. 3D). To interpret the biological significance of these genes within a structured functional framework, GO enrichment analysis was performed for the 521 DEGs across three categories: Biological Process (BP) category, Cellular Component (CC) category (Supplementary Fig. 3), the Molecular Function (MF) category (Supplementary Fig. 4). Under the Biological Process (BP) category, the consolidated GO terms highlighted key concepts such as "response," "homeostasis","metabolic" and "apoptotic", indicating a potential disturbance in essential cellular activities (Fig. 3E). The KEGG pathway annotation was refined to enable the identification of significant pathways associated with EVO (Fig. 3F), predominantly including the PPAR pathway, TNF pathway, PI3K-Akt pathway, NF-κB pathway, tight junction (TJ) pathway, and the apoptosis pathway. To validate the critical regulatory nodes within the PPARα/NF-κB/TJ/Apoptosis signaling cascade, western blot analyses were conducted for key molecular markers including PPARα, p-P65, Occludin, ZO-1, and Caspase-3. The results revealed that, in comparison with the Ctrl, the expression levels of PPARα, p-P65, ZO-1, and Caspase-3 were markedly elevated in a dose-dependent manner (*p* < 0.05), while Occludin expression was significantly suppressed (*p* < 0.01) (Fig. 3F and G). These observations indicate that EVO modulates the PPARα/NF-κB/TJ/Apoptosis pathway, leading to impaired tight junction integrity and disruption of cellular polarity. This disturbance ultimately interferes with bile acid secretion and triggers cytotoxic effects in cells.

### EVO-induced hepatotoxicity in mice

**Fig. 4:**
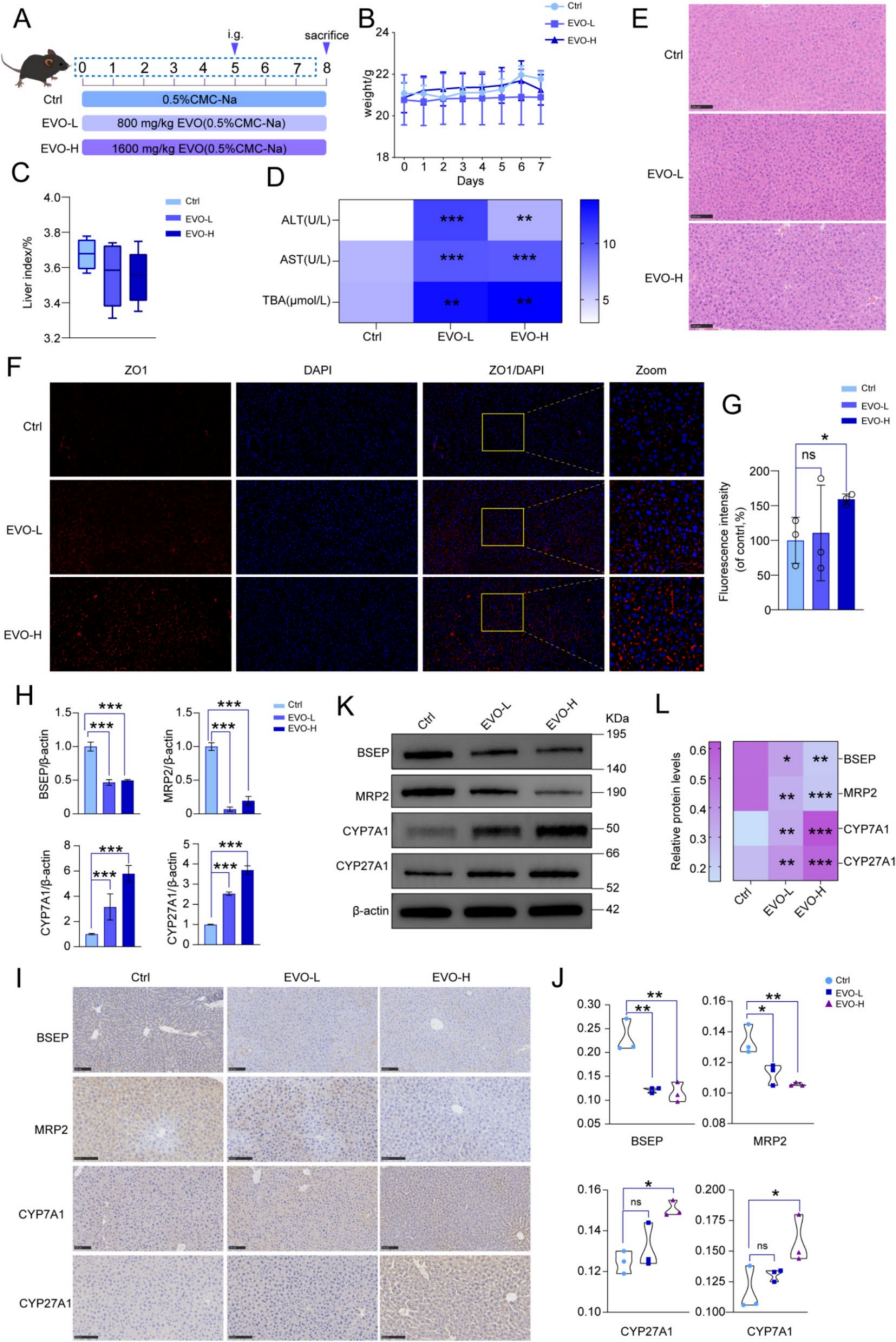
Hepatotoxicity of EVO in mice. **A** The experimental schedule of EVO treatment in mice. **B** Daily weight of mice in each group. **C** The liver index. **D** The levels of ALT, AST and TBA. **E** HE staining of liver tissue sections. **F**-**G** The immuno-fluorescence results for ZO-1 protein in mice liver tissue. **H** The results of qPCR for BSEP, MRP2, CYP27A1, and CYP7A1 genes in mice liver tissue. **I**–**L** The immunohistochemical (**I**-**J**) and western blot (**K**–**L**) results for BSEP, MRP2, CYP27A1, and CYP7A1 proteins in mice liver tissue. Data are expressed as means ± SD (n = 6 per group for weight and liver index; n = 3 per group for ALT, AST, TBA, qPCR, western blot, immunohistochemistry and immunofluorescence). ns, non-significant; *P < 0.05; **P < 0.01; ***P < 0.001

Further observations were meticulously carried out to evaluate the hepatic function in mice following EVO administration (Fig. 4A). Compared to the Ctrl group, mice treated with EVO-L and EVO-H exhibited a noticeable decline in body weight by Day 6 (Fig. 4B), accompanied by a mild reduction in liver index ((Liver wet weight / Body weight) × 100%) (Fig. 4C), as well as marked increases in serum ALT, AST, and TBA levels (*p* < 0.01) (Fig. 4D). Histopathological analysis revealed evident hepatocyte swelling, blurred nuclear margins, increased inflammatory cell infiltration, and prominent accumulation of intracellular lipid droplets (Fig. 4E). Collectively, these findings suggest that EVO administration leads to substantial liver injury and functional deterioration in mice. Moreover, following EVO intervention, a pronounced upregulation in the expression of ZO-1 was detected in hepatic tissues (*p* < 0.05) (Fig. 4F and G). In parallel, integrated analysis of RT-PCR analyses, western blot and immunohistochemical revealed significant alterations in the expression profiles of genes implicated in BAs synthesis and transport in EVO-treated mice. Notably, crucial efflux transporters such as BSEP and MRP2 exhibited substantial downregulation upon EVO exposure. Concurrently, a marked increase was observed in the expression levels of key BA biosynthetic enzymes, namely CYP27A1 and CYP7A1 (*p* < 0.001) (Fig. 4H–L), suggesting a potential disturbance in BA metabolic homeostasis, which may culminate in elevated BA accumulation. These in vivo and in vitro observations collectively imply that EVO may compromise TJ integrity, thereby interfering with the physiological processes of BA synthesis and transport, ultimately predisposing to cholestatic injury.

### EVO disrupts BAs homeostasis in the livers of mice.

**Fig. 5:**
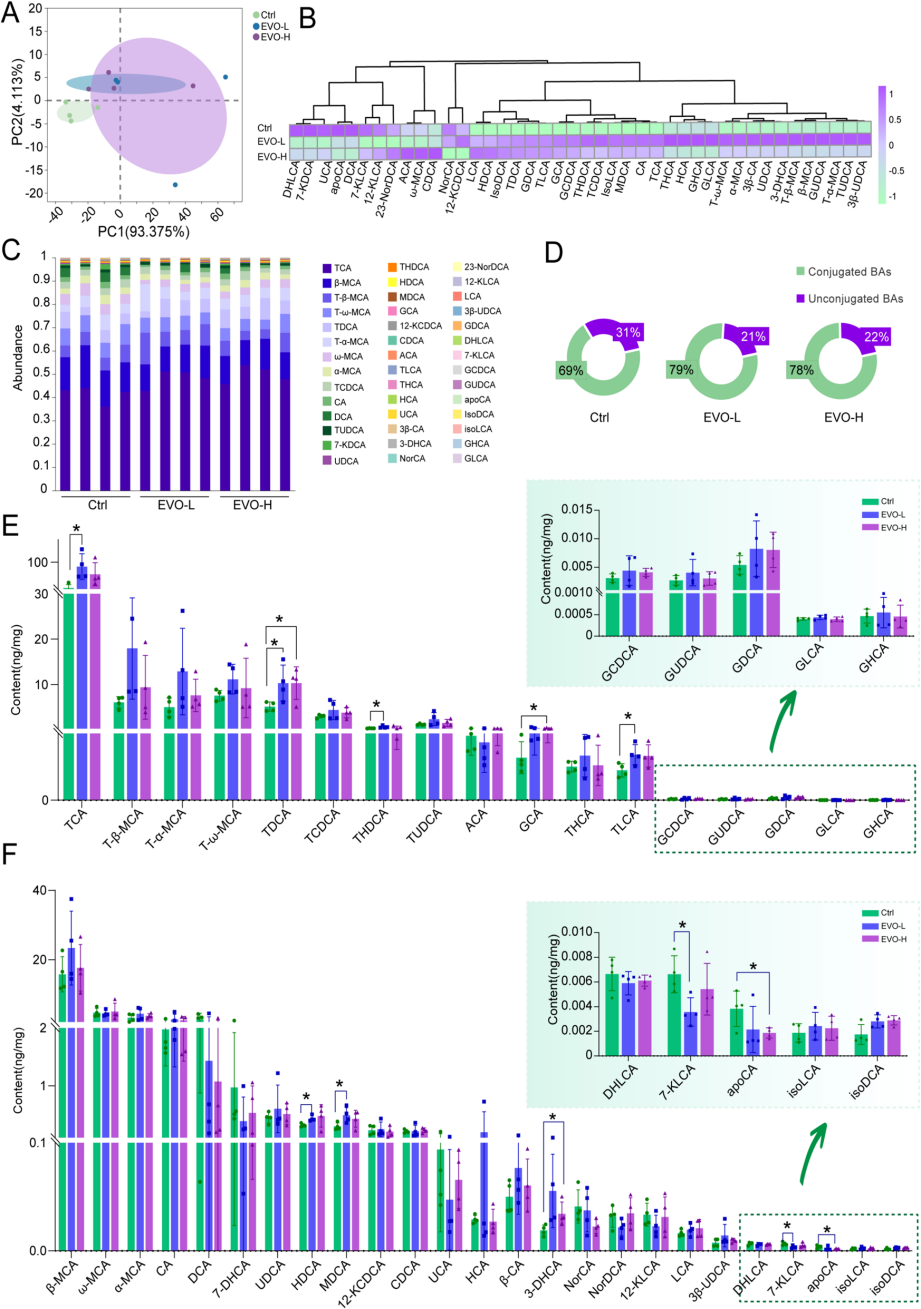
The effect of EVO on BAs metabolism in mice liver. **A** PCA score plots. **B** The clustered heatmap of the levels of 47 BAs in the livers of mice from three groups. **C** The ratio of total conjugated BAs to total unconjugated BAs. **D** The abundance of 47 BAs within samples from three groups. **E** The content of conjugated BAs within samples from various groups. **F** The content of unconjugated BAs within samples from various groups. Data are expressed as means ± SD (n = 4 per group). *P < 0.05

Further targeted detection was conducted to investigate the effect of EVO on BA metabolism in the liver, with particular emphasis on 47 different BAs. To comprehensively characterize the overall metabolic profiles of BAs across different treatment groups, PCA was initially employed to systematically identify and summarize the primary intergroup variations. The results revealed distinct differences in the global metabolic patterns among the sample groups. Alterations in hepatic BAs levels may impair normal physiological functions of the liver. Elevated BAs concentrations can disrupt fat digestion, leading to clinical manifestations such as malabsorption and diarrhea. BAs also play a critical role in modulating multiple signaling pathways in the liver, influencing hepatocyte proliferation, differentiation, and apoptosis. Excessive accumulation of BAs may exert cytotoxic effects on hepatocytes and trigger inflammatory responses, with prolonged exposure contributing to the development of liver pathologies, including cholestasis and cirrhosis. Conversely, abnormally levels of BAs may compromise bile secretion and excretion, thereby impairing the liver's metabolic and detoxification capacities (Fig. 5A). Additionally, the heatmap illustrated the detailed changes in hepatic BAs content, along with elevated levels of multiple BAs following EVO (Fig. 5B). Meanwhile, the proportions change of individual BAs were manifested after EVO administration (Fig. 5D). Furthermore, EVO treatment resulted in an increased proportion of conjugated BAs and a concomitant decrease in free BAs (Fig. 5C). Specifically, significant elevations were noted in conjugated BAs such as TCA, TDCA, THDCA, TLCA, and GCA (*p* < 0.05) (Fig. 5E). Conversely, a marked reduction was evident in the levels of unconjugated BAs, such as HDCA and MDCA (*p* < 0.05) (Fig. 5F).

**Fig. 6:**
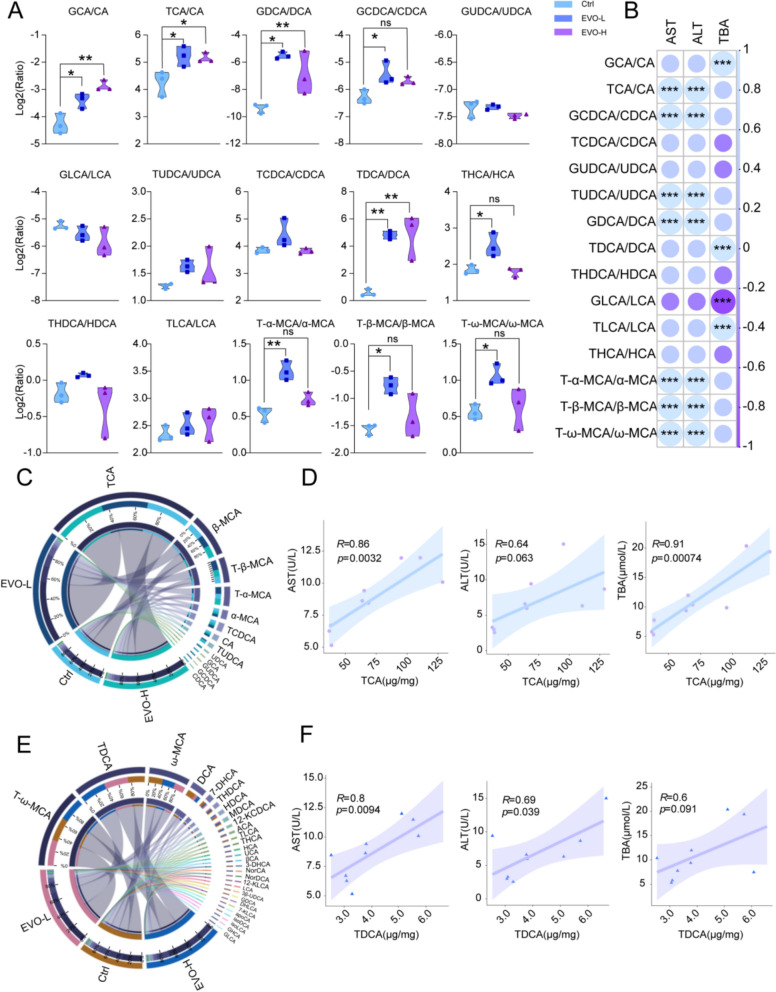
Specific changes in the ratio of conjugated to unconjugated BAs. **A** The ratios of 15 pairs of conjugated to unconjugated BAs. **B** The correlations of 15 pairs of conjugated to unconjugated BAs with ALT, AST and TBA. **C** The impact of EVO on primary BAs in the liver. **D** The correlation of primary BAs changes with liver injury indicators. **E** The impact of EVO on secondary BAs in the liver. **F** The correlation of secondary BAs changes with liver injury indicators. Data are expressed as means ± SD (n = 3 per group). ns, non-significant; *P < 0.05; **P < 0.01; ***P < 0.001

Furthermore, by comparing the ratios of 15 pairs of conjugated to unconjugated BAs, it was discovered that EVO modified the ratios of conjugated to unconjugated BAs to a certain extent, including GCA/CA, TCA/CA, GDCA/DCA, GCDCA/CDCA, TDCA/DCA, THCA/HCA, T-α-MCA/α-MCA, T-β-MCA/ β-MCA, and T-ω-MCA/ω-MCA (Fig. 6A). Notably, changes in the ratios of TCA/CA, GCDCA/CDCA, TUDCA/UDCA, GDCA/DCA, and several MCA BAs displayed strong correlations with transaminase levels in mice. Meanwhile, the variations in the ratios of GCA/CA, TDCA/DCA, GLCA/LCA, and TLCA/LCA demonstrated strong correlations with total BAs levels in mice (Fig. 6B). BAs are primarily synthesized in the liver and transported to the intestine through the biliary system. The majority of BAs are reabsorbed in the terminal ileum and returned to the liver through enterohepatic circulation, while the unabsorbed fraction enters the colon, where they undergoes bacterial metabolism to produce secondary BAs. Therefore, the effects of EVO on primary and secondary BAs in the liver was further illustrated (Fig. 6C and E). Additionally, among the BAs analyzed, TCA (a primary BAs) and TDCA (a secondary BAs) exhibited the most pronounced alterations following EVO-induced cholestasis. The changes in the concentrations of these two BAs also exhibited a positive correlation with established liver injury markers (Fig. 6D and F). Therefore, these results indicate that EVO-induced perturbations in the bile acid metabolic profile contribute to hepatic injury, primarily through the accumulation of BAs due to disrupted synthesis and metabolic balance. Specifically, the ratios of TCA/CA, GCDCA/CDCA, TUDCA/UDCA, GDCA/DCA, GCA/CA, TDCA/DCA, GLCA/LCA, and TLCA/LCA, as well as the contents of TCA and TDCA, may serve as potential indicators of the severity of EVO-induced cholestasis to a certain extent. These findings provide evidence that EVO disrupts BAs homeostasis in the livers of mice.

### Different manifestations of EVO induced hepatotoxicity in healthy and UC individuals

**Fig. 7:**
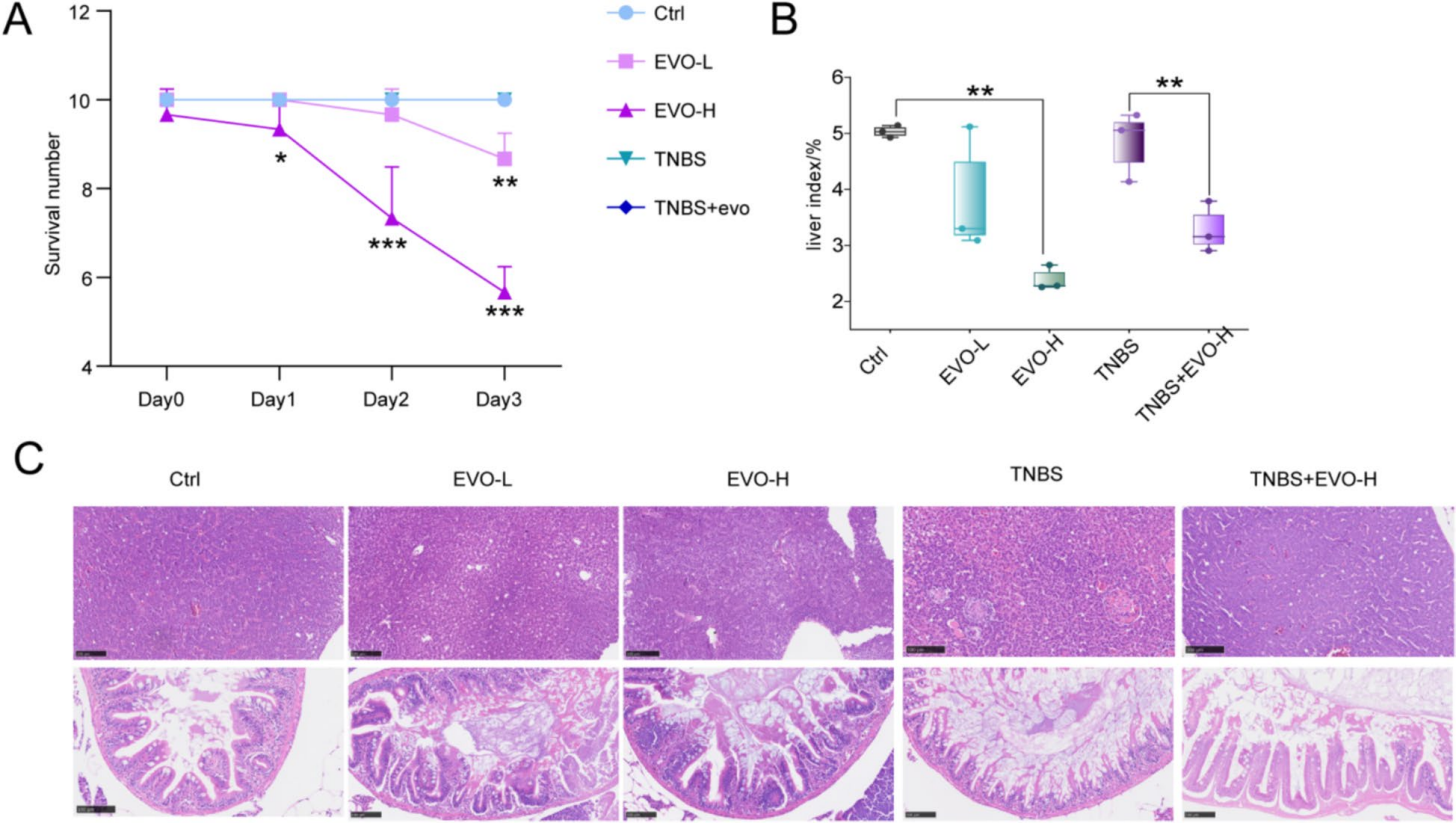
The hepatotoxicity of EVO on healthy and TNBS-induced IBD zebrafish. **A** The survival number under EVO exposure. **B** Liver index of zebrafish. **C** The result of HE staining for liver and colon tissues of zebrafish. Data are expressed as means ± SD in the liver index (n = 3 per group for liver index and HE staining). ns, non-significant; **P < 0.01

To compare the different manifestations of EVO on the livers of individuals with and without UC, zebrafish in distinct physiological states were subjected to identical concentrations of EVO. The result revealed that adult zebrafish under UC conditions exhibited a markedly enhanced tolerance to EVO. Specifically, exposure to EVO-L for 48 h resulted in significant mortality in zebrafish, whereas exposure to EVO-H for 24 h induced a pronounced lethal effect. Moreover, the mortality rate in the EVO-H group was significantly higher than that in the EVO-L group among healthy zebrafish. Notably, however, no mortality was observed in the TNBS + EVO-H treatment group (Fig. 7A). Furthermore, EVO induced pronounced hepatotoxic effects in healthy zebrafish, culminating in a substantial reduction in the liver index (*p* < 0.01) (Fig. 7B). Histological analysis demonstrated that TNBS administration could compromise intestinal architecture without inducing substantial hepatic injury. Notably, EVO treatment not only ameliorated the intestine structural damage caused by TNBS but also exhibited minimal hepatotoxicity when compared to healthy zebrafish (Fig. 7C).

**Fig. 8:**
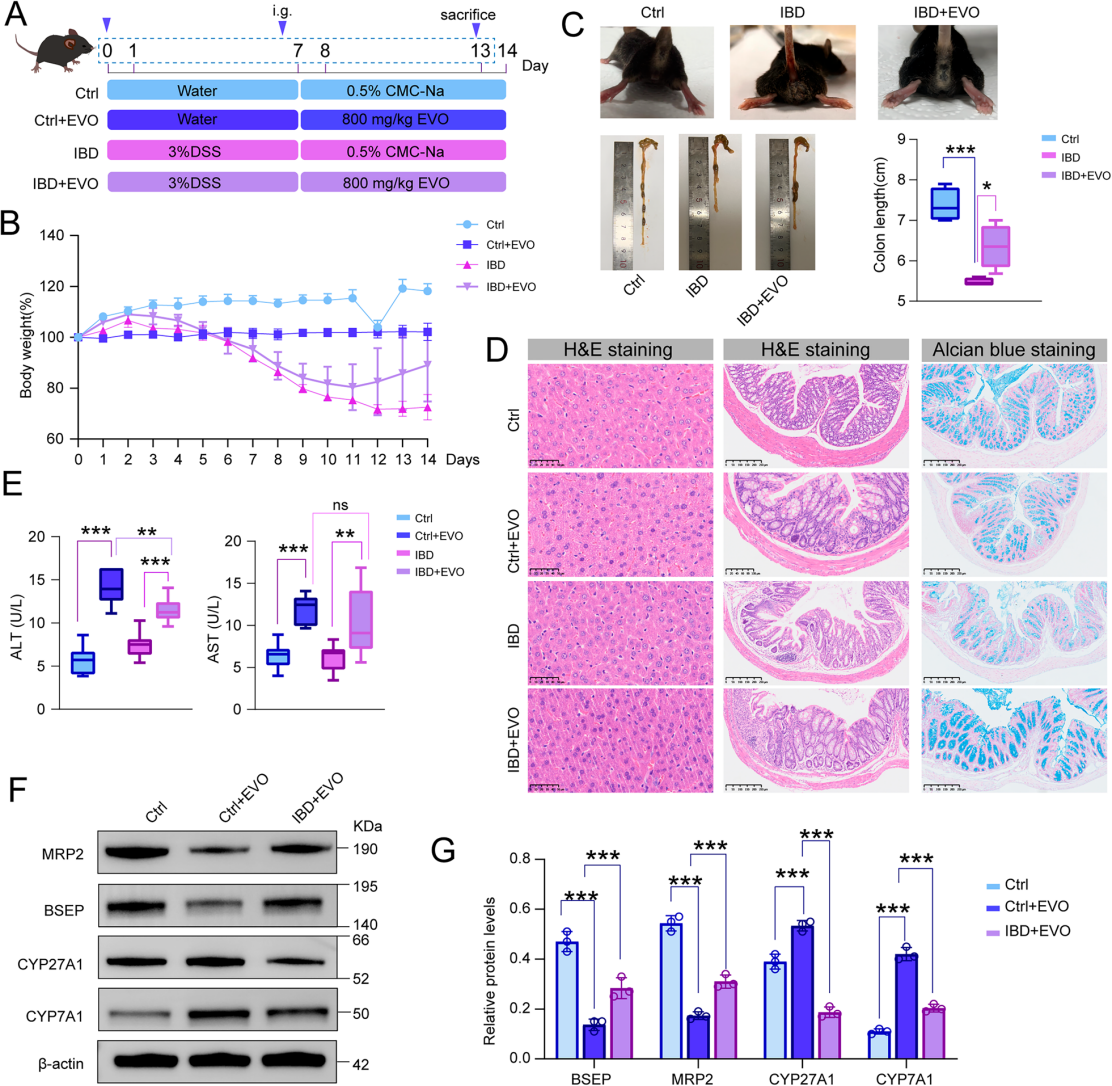
The effect of EVO on the intestines and liver of healthy and IBD mice. **A** The experimental schedule of EVO treatment in healthy and IBD mice. **B** Daily weight of mice in each group. **C** The ameliorative effect of EVO on intestinal symptoms in IBD mice. **D** HE staining of liver tissue sections and HE staining as well as Alcian blue staining of colon tissue sections. **E** The levels of ALT and AST. **F**–**G** The western blot results for BSEP, MRP2, CYP27A1, and CYP7A1 proteins in mice liver tissue. Data are expressed as means ± SD (n = 6 per group for body weight and colon length; n = 3 per group for ALT, AST and western blot). ns, non-significant; *P < 0.05; **P < 0.01; ***P < 0.001

In the murine model, compared with the Ctrl group, mice in the Ctrl + EVO group exhibited no remarkable alterations in body weight. Mice with induced UC continued to display progressive weight loss, whereas those in UC + EVO experienced initial weight loss followed by a gradual recovery (Fig. 8B). The colon length in UC mice was markedly shortened relative to the Ctrl, accompanied by intestinal hemorrhage (P < 0.001) (Fig. 8C). Histopathological analysis revealed that UC mice exhibited villous atrophy, disrupted mucosal epithelium, decreased goblet cell density, lymphocyte and granulocyte infiltration, as well as occasional lymph node formation (Fig. 8D). Furthermore, Alcian blue staining demonstrated that the secretory function of goblet cells was significantly impaired in UC mice, as evidenced by decreased mucin production (Fig. 8D). Remarkably, these pathological manifestations were markedly ameliorated following EVO treatment, underscoring its therapeutic potential in ameliorating UC-related symptoms. More importantly, the hepatotoxic effects associated with EVO administration were significantly attenuated in UC mice compared to healthy Ctrl, as demonstrated by lower serum levels of ALT and AST (*p* < 0.01) (Fig. 8E), along with improved hepatic histological structure (Fig. 8D). Strikingly, EVO treatment in UC mice restored the expression levels of key hepatic transporters and enzymes-including BSEP, MRP2, CYP7A1, and CYP27A1-to levels comparable to those in the control group (*p* < 0.05) (Fig. 8F and G). Collectively, these findings from both zebrafish and mice models vividly illustrate that EVO possesses the remarkable ability to mitigate the symptoms of UC and ameliorate the pathological alterations in the intestinal barrier. Moreover, EVO exhibits a markedly diminished level of hepatotoxicity in individuals with UC, underscoring its potential as a safer therapeutic option in this patient population.

### The dynamic along the gut-liver axis contributes to the transformation of toxicity into efficiency

**Fig. 9:**
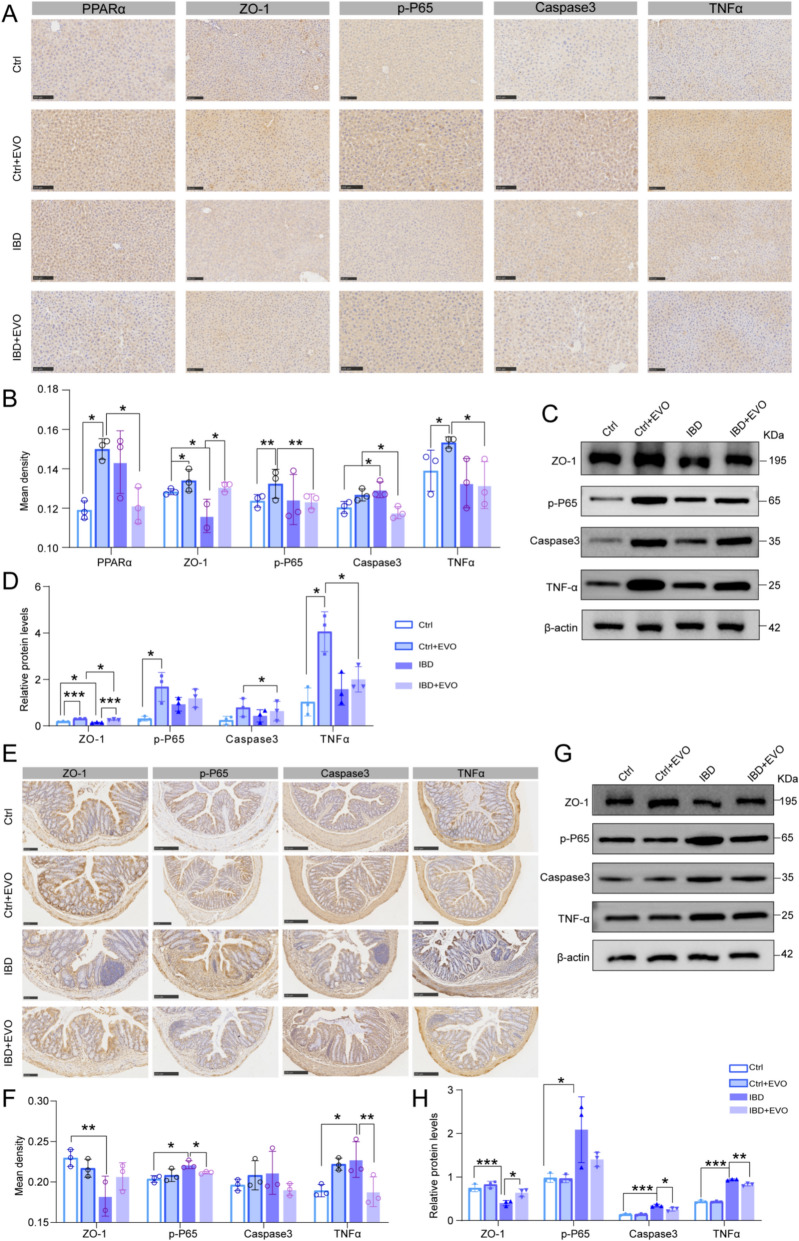
The regulatory effects of EVO on several key proteins in the liver and colon of both healthy and IBD mice. (**A**–**D**) Immunohistochemical staining (A, B) and western blot (**C**, **D**) results for PPARα, ZO-1, TNFα, p-P65, and Caspase3 proteins in liver tissues. (E**–**H) Immunohistochemical staining (**E**, **F**) and western blot (**G**, **H**) results for ZO-1, TNFα, p-P65, and Caspase3 proteins in colon tissues. Data are expressed as means ± SD (n = 3 per group). ns, non-significant; *P < 0.05; **P < 0.01; ***P < 0.001

To investigate this differential expression of key proteins associated with EVO-induced hepatotoxicity, we assessed the expression levels of ZO-1, TNFα, p-P65, and Caspase-3 in both liver and colon tissues of experimental mice. Immunohistochemical staining and western blot analyses demonstrated that EVO administration significantly enhanced the expression of PPARα, TNFα, p-P65, and Caspase-3 in the liver of normal mice. Notably, in the IBD + EVO group, the expression levels of these proteins were markedly reduced compared to those in the EVO group (P < 0.05) (Fig. 9 A-D). Intriguingly, EVO exerted minimal influence on the expression of these proteins in the colons of healthy mice; however, it effectively ameliorated the abnormal alterations in these protein levels observed in the colons of UC-affected mice (p < 0.05) (Fig. 9 E–H). Collectively, these findings underscore a distinct divergence in the expression profiles of ZO-1, TNFα, p-P65, and Caspase-3 with the liver-gut axis under healthy and UC conditions, contributing to the bidirectional regulatory effect of EVO. These results further imply that the strategic application of EVO not only mitigates potentially adverse effects but also enhances its therapeutic mechanisms, potentially transforming adverse reactions into beneficial outcomes.

## Discussion

This study systematically evaluated the hepatotoxicity profile of EVO through in vitro experiments and in vivo animal models. By employing an integrated multi-omics approach, it was confirmed that EVO induces cholestasis by disrupting bile acid metabolism and inflammatory pathways, thereby causing hepatotoxicity in healthy organisms. However, in the context of IBD model, EVO not only demonstrated significant therapeutic efficacy against IBD but also exhibited a marked reduction in hepatotoxic potential. Further investigations suggested that dynamic alterations in the PPAR/TJ/apoptosis pathway along the gut-liver axis under varying physiological conditions may constitute a key mechanism underlying the attenuation of EVO-induced toxicity, the maintenance of its therapeutic efficacy, and potentially the conversion of adverse effects into beneficial outcomes.

### The hepatotoxicity of EVO is associated with the disruption of BAs metabolism

As a widely utilized TCM for treating gastrointestinal disorders, the application of Evodia rutaecarpa can be traced back to the "Shennong's Classic of Materia Medica". Contemporary research has identified EVO as a crucial bioactive component in Evodia rutaecarpa responsible for its therapeutic effects on UC, through potential modulation of multiple biological pathways, such as the regulation of microbial homeostasis [9]. Given the growing demand for natural and sustainable therapies, EVO presents considerable promise for further development [22]. However, emerging evidence of EVO-induced toxicity has raised concerns that limit its clinical application and hinder the advancement of novel therapeutics. Prior studies have demonstrated that EVO can induce multi-organ damage, with particular emphasis on hepatotoxicity [4]. However, inconsistencies across these studies have impeded the establishment of a standardized framework for comprehensive toxicological evaluation of EVO. Therefore, the development of a reliable evaluation model that accurately reflects the toxicological characteristics of EVO [23] coupled with systematic investigation into its hepatotoxic manifestations and underlying mechanisms through modern biotechnology, represents the critical and distinctive strategy for addressing the current obstacles associated with EVO‘s safe and effective utilization.

Consequently, we utilized conventional in vitro cell models and in vivo models to further investigate the hepatotoxic effects of EVO. The results demonstrated that EVO-induced hepatotoxicity, observed across cell lines, mice, and zebrafish, was characterized by dose-dependent disruption of liver cell morphology, impaired liver function, and significant cell apoptosis.

To accurately characterize the hepatotoxicity of EVO, we further employed non-targeted metabolomics in cellular models and targeted BAs metabolomics in mice to systematically elucidate the profile of endogenous metabolites in EVO-treated samples. These findings revealed remarkable disruptions in BAs metabolism, as evidenced by alteration of types and proportions of BAs. Furthermore, Western blot results demonstrated that administering EVO to healthy individuals introduced a complex interplay affecting BA homeostasis. On one hand, EVO elicited a downregulation of hepatic BA transporters-BSEP, located on the canalicular membrane, and MRP2, prominently positioned on the apical membrane of hepatocytes. Researchers have documented that both BSEP and MRP2 play vital roles in BA excretion, with BSEP facilitating canalicular efflux and MRP2 orchestrating the efficient efflux of BAs and their conjugates from hepatocytes into bile, thereby safeguarding the delicate balance of BAs [24, 25]. This suppression in transporter expression subsequently impeded the excretion of BAs from hepatocytes. On the other hand, EVO stimulated BA synthesis by potentiating the expression levels of CYP27A1 and CYP7A1 [26], which are indispensable enzymes within the BA synthesis pathway, responsible for catalyzing the formation of BAs. Consequently, these disruptions result in a significant increase in intracellular TBA levels, disrupting the tightly regulated homeostasis of the BAs pool within liver tissue, thereby leading to hepatotoxicity.

### Disruption of hepatocyte polarity and inflammation, mediated by BAs metabolic disorder, further exacerbate the hepatic toxicity induced by EVO

To achieve a more comprehensive understanding of the hepatotoxicity induced by EVO, we employed transcriptomic techniques to examine the impact of EVO on the global transcriptional profile of hepatic cells. The result substantiated that the EVO-induced hepatotoxicity involves multiple biological processes, including BAs imbalance, inflammatory response, and hepatocyte apoptosis.

Extensive research has demonstrated that BAs metabolism disorders in hepatic cells are closely associated with inflammatory reactions and cellular apoptosis [27]. Specifically, accumulated BAs can activate downstream NF-κB inflammatory response and upregulate TNF-α expression [28]. TNF-α, a proinflammatory cytokine, is elevated during inflammation and contributes to lipid peroxidation and oxidative stress, while also inducing the release of additional inflammatory mediators, thereby disrupting the balance of inflammatory response. The NF-κB protein family, a transcription factor complex, is involved in regulating the expression of diverse inflammatory genes. In addition, TNF-α and NF-κB possess the capacity to mutually activate each other, establishing a detrimental cycle that exacerbates inflammation. Upon stimulation by cytokines such as TNF-α, the inhibitor of κB kinase (IKK) complex dissociates into its subunits IKK1/IKKα and IKK2/IKKβ, leading to the activation of the NF-κB pathway and the subsequent release of proinflammatory cytokines, such as IL-6, IL-1β, and TNF-α. Thus, administration of EVO was found to increase the levels of TNF-α and NF-κB p65 in liver, confirming the presence of an inflammatory response.

The inflammation triggered by BAs can further result in cellular apoptosis [29]. Cell apoptosis mainly occurs through two distinct pathways: the extrinsic pathway, which involves the activation of caspases via extracellular signals and death receptor engagement [30], and the intrinsic pathway, mediated by the release of apoptotic factors from mitochondria [31]. TNF-α functions as a ligand that activates death receptors, thereby inducing apoptosis, as evidenced by elevated Caspase-3 levels observed in cellular and mouse liver models, corroborated by flow cytometry results. Nevertheless, hepatic cells appear to initiate a protective response following EVO-induced liver injury. The farnesoid X receptor (FXR), a major receptor of BAs, plays a critical role not only in maintaining BAs homeostasis [32], but also in regulating lipid and glucose metabolism [33]. Studies have demonstrated that BAs can activate FXR, which subsequently leads to the upregulation of peroxisome proliferator-activated receptor alpha (PPARα). PPARα serves as a pivotal regulator in BAs metabolism and glucuronidation by modulating the expression of cytochrome P450 (CYP) and uridine 5'-diphospho-glucuronosyltransferase (UGT) enzymes [34]. Additionally, it exhibits inhibitory effects on pro-inflammatory and acute phase response signaling pathways [35]. In both cellular and mouse liver models, EVO intervention has been associated with elevated levels of PPARα, likely-mediated through BA-induced activation of FXR. This disruption subsequently triggers inflammation responses, cell apoptosis, and ultimately culminating in liver injury.

### The dynamic modification of the hepato-intestinal axis could potentially serve as a crucial factor in elucidating the alterations in the toxic effects of EVO

The maintenance of gut-liver axis homeostasis has emerged as a prominent focus within contemporary biomedical research. Extensive studies have substantiated the intimate association between the gut-liver axis and the pathogenesis, progression, and clinical outcomes of various hepatic [36] and gastrointestinal disorders [37]. Furthermore, this bidirectional axis is increasingly recognized as a critical pathway and therapeutic target for the pharmacological management of these conditions [38]. Nevertheless, current literature remains limited regarding the role of gut-liver axis homeostasis in the transformation of drug toxicity and efficacy.

The TJ protein plays a crucial role in sustaining the polarized architecture of hepatocytes and regulating BAs metabolism. Additionally, it constitutes an essential element in preserving the integrity of the intestinal barrier [39], preventing bacterial translocation, and facilitating mucosal repair following damage [40]. Consequently, the TJ protein has garnered significant attention in the evaluation of therapeutic agents for intestinal disorders [41, 42]. Recently, there has been extensive research exploring the therapeutic effects of EVO in the treatment of UC [3]. In addition, consistency was observed in the alteration of TJ protein level along the gut-liver axis [43]. Individuals with UC exhibit decreased TJ protein levels both in the intestine and the liver [44]. Notably, UC can significantly alleviate cholestasis through the inhibition of BAs synthesis [45]. Based on these findings, we propose the following hypothesis: The hepatotoxic effects and therapeutic benefits of EVO in treating UC may both be mediated through its regulation of some proteins; moreover, while the differential hepatotoxicity induced by EVO could be associated with variations in target protein expression between individuals and those with UC. Thus, we established an UC mouse model to test this hypothesis.

Fortunately, the comparative analysis between healthy animals and those with UC revealed that the EVO-induced hepatotoxicity was alleviated in the UC model, characterized by diminished alterations in liver tissue structure and liver function enzyme levels. Furthermore, compared with the Ctrl group, the key proteins associated with BAs metabolism (BSEP, MRP2, CYP27A1 and CYP7A1), inflammation (TNF-α, NF-κB and p-P65) and apoptosis (Caspase-3) exhibited distinct patterns in the livers of both normal and UC mice. More importantly, administration of EVO to UC subjects resulted in a significant increase in TJs (ZO-1), but marked decrease in Caspase-3, TNF-α, NF-κB and p-P65 levels in the colon induced by DSS. Notably, these effects were observed specifically in the colon but not in the liver of UC models, where no significant differences were detected. These observations not only demonstrated the efficacy of EVO in treating UC, but also confirmed the mitigated hepatotoxicity of EVO under UC conditions UC. It suggests the biological response to EVO is modulated according to the physiological changes along the gut-liver axis in both normal and pathological states.

### The investigation into the toxic effects of EVO can contribute to the advancement of clinical precision medicine and the development of EVO-based therapies

Toxic TCM plays an integral role in TCM clinical practice. However, several challenges impede its broader application, including underreporting of adverse events, increasing concerns regarding drug safety, and the lack of a clear correlation between toxicity and therapeutic efficacy. Notably, the concept of toxicity in TCM theory fundamentally differs from that in Western medicine, as it emphasizes the pathological state of the organism rather than intrinsic chemical toxicity. Therefore, the toxicity assessment should be conducted within the context of clinical application, rather than relying exclusively on high-dose, long-term administration in normal animals. Importantly, TCM perceives herb toxicity as an inherent regulatory mechanism governing the drug‘s activity toward specific biological events, as illustrated by the principles of "Yidu Gongdu" and "Yougu Wuyun". Consequently, it is essential to systematically investigate the distinct toxicity characteristics of different toxic herbs medicines and establish the relationship between toxicity and efficacy. This underscores the importance of exploring the underlying toxic mechanisms of these agents and highlights the necessity of precision medicine-ensuring both the therapeutic efficacy and safety of medicines through appropriate dosing tailored to individual disease conditions [46].

Therefore, building upon prior research, this study further elucidated the mechanistic association between EVO-induced hepatotoxicity and BAs metabolism, inflammatory responses, and cellular apoptosis, thereby providing a theoretical foundation for a deeper understanding of the scientific basis underlying processing methods and drug compatibility in mitigating EVO‘s hepatotoxic effects. Furthermore, integrating the clinical application context of EVO with the principles of TCM theory, a comprehensive model characterizing the relationship between its hepatotoxicity and therapeutic efficacy in UC was established. This work transcends the conventional reliance on high-dose and long-term administration paradigms in hepatotoxicity assessment. Notably, it innovatively incorporates physiological state-related factors that influence EVO-induced hepatotoxicity, emphasizing the pivotal role of liver-gut axis homeostasis in modulating both drug-induced liver injury and pharmacological activity. Collectively, this finding offers valuable insights for the subsequent precise development of EVO and other TCM-derived compounds. In future evaluations of the hepatotoxicity of traditional Chinese medicine, greater consideration should be given to the unique characteristics of clinical drug use environments. Building upon modern biological theories and advanced technologies, a comprehensive and objective assessment of drug safety should be conducted by integrating the dynamic interplay and intrinsic relevance between toxicity and pharmacodynamic effects.

Additionally, EVO may contribute to the restoration of key proteins in the liver and intestine, suggesting that these proteins could serve as potential therapeutic targets for UC or be involved in related pathophysiological mechanisms. However, the precise molecular mechanisms underlying EVO's action and its interactions with target proteins remain unclear. Moreover, what is the optimal balance between therapeutic efficacy and hepatotoxicity when administering EVO for the treatment of UC? Clearly, further in-depth research is warranted to address these critical questions.

## Conclusion

**Fig. 10:**
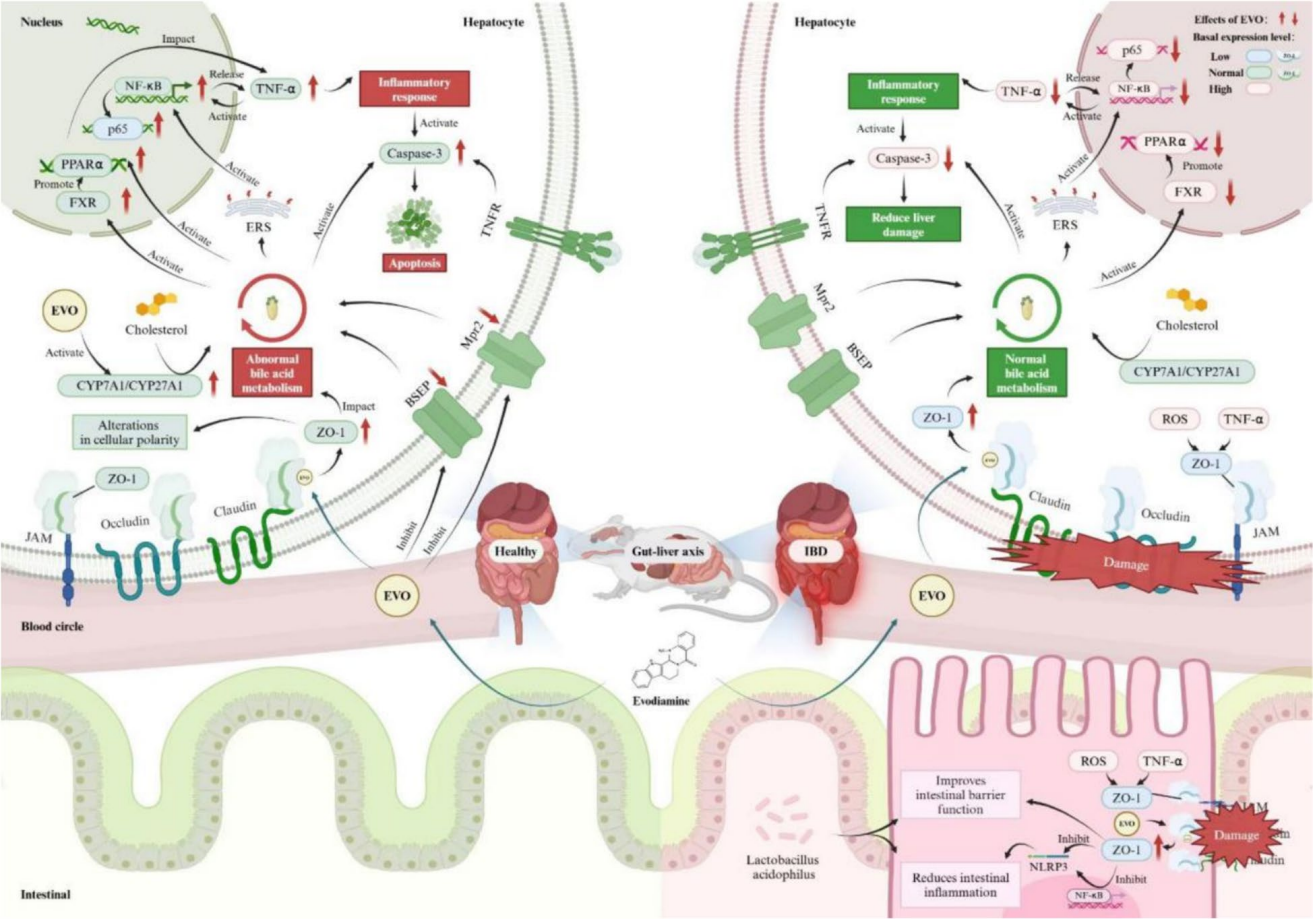
The molecular mechanism of transformation of hepatotoxicity and efficacy of EVO against inflammatory bowel disease

In summary, the different expression levels of PPAR/NF-κB/ZO-1/caspase-3 pathway within the gut-liver axis constitute the central mechanism underlying the dual effects of EVO in treating UC and inducing liver injury (Fig. 10). These findings not only confirm the intimate connection between gut-liver axis homeostasis and drug toxicity transformation, but also provide robust empirical evidence supporting the principles of "Yidu Gongdu" and "Yougu Wuyun" in TCM. Furthermore, they offer novel insights for the precise development and safety optimization of EVO and other potentially toxic herbal medicines.

## Supplementary Information

Additional file 1.

## Data Availability

No datasets were generated or analysed during the current study.

## Abbreviations

ALT: Alanine transaminase
AST: Aspartate aminotransferase
BA: Bile acid
BP: Biological process
BSEP: Bile salt export pump
CA: Cholic acid
CC: Cellular component
CCK8: Cell counting kit-8
CD: Crohn’s disease
CDCA: Chenodeoxycholic acid
Ctrl: Control
CYP27A1: Cytochrome P450 Family 27 Subfamily A Member 1
CYP7A1: Cytochrome P450 Family 7 Subfamily A Member 1
DCA: Deoxycholic acid
DEPC: Diethylpyrocarbonate
DMSO: Dimethyl sulfoxide
DSS: Dextran sodium sulfate
ECL: Enhanced chemiluminescence
EDTA: Ethylenediaminetetraacetic acid
ERS: Endoplasmic reticulum stress;
EVO: Evodiamine
EVO-H: High dose of evodiamine
EVO-L: Low dose of evodiamine
FITC: Fluorescein isothiocyanate
FXR: Farnesoid X receptor
GAPDH: Glyceraldehyde-3-phosphate dehydrogenase
GCA: Glycocholic acid
GCDCA: Glycochenodeoxycholic acid
GDCA/DCA: Ratio of glycodeoxycholic acid to deoxycholic acid
GLCA: Glyco-lithocholic acid
GLCA/LCA: Ratio of glycolithocholic acid to lithocholic acid
GO: Gene ontology
GCA/CA: Ratio of glycocholic acid to cholic acid
HDCA: Hydroxycholic acid
HE: Hematoxylin & eosin
HRP: Horseradish peroxidase
IBD: Inflammatory bowel disease
IKK: Inhibitor of κB kinase
IL-1β: Interleukin-1 beta
IL-6: Interleukin-6
LCA: Lithocholic acid
MDCA: Muricholic acid
MF: Molecular function
MRP2: Multidrug resistance-associated protein 2
NF-κB: Nuclear factor kappa B
OPLS-DA: Orthogonal partial least squares discriminant analysis
PBS: Phosphate buffered saline
PCA: Principal component analysis
PCR: Polymerase chain reaction
PI: Propidium iodide
PLS-DA: Partial least squares discriminant analysis
PPAR: Peroxisome proliferator-activated receptor
PVDF: Polyvinylidene fluoride
qPCR: Quantitative polymerase chain reaction
SDS-PAGE: Sodium dodecyl sulfate–polyacrylamide gel electrophoresis
SE: Simplify enrichment
T-α-MCA: Tauro-α-muricholic acid
T-β-MCA: Tauro-β-muricholic acid
T-ω-MCA: Tauro-ω-muricholic acid
TBA: Total bile acid
TC: Total cholesterol
TDCA: Taurodeoxycholic acid
TDCA/DCA: Ratio of taurodeoxycholic acid to deoxycholic acid
TG: Triglyceride
THDCA: Tauro-α-muricholic acid
TJ: Tight Junction
TLCA: Tauro-β-muricholic acid
TLCA/LCA: Ratio of tauro-lithocholic acid to lithocholic acid
TCA: Taurocholic acid
TCA/CA: Ratio of taurocholic acid to cholic acid
TUDCA: Tauroursodeoxycholic acid
TUDCA/UDCA: Ratio of tauroursodeoxycholic acid to ursodeoxycholic acid
UC: Ulcerative colitis
UDCA: Ursodeoxycholic acid
UGT: Uridine 5'-diphospho-glucuronosyltransferase
VIP: Variable importance in projection
ZO-1: Zonula occludens-1
