# Supplementary material for: The conversion of evodiamine-induced hepatotoxicity into a therapeutic effect on colonitis: insight from the liver-gut axis mediated by PPAR/NF-κB/ZO-1/caspase-3 pathway
Source: Chin Med. 2025 Nov 21;20:198. doi: 10.1186/s13020-025-01262-3 (PMC12636192; doi:10.1186/s13020-025-01262-3)
Supplement: Supplementary file 1 — Additional file 1. [file 13020_2025_1262_MOESM1_ESM.docx]

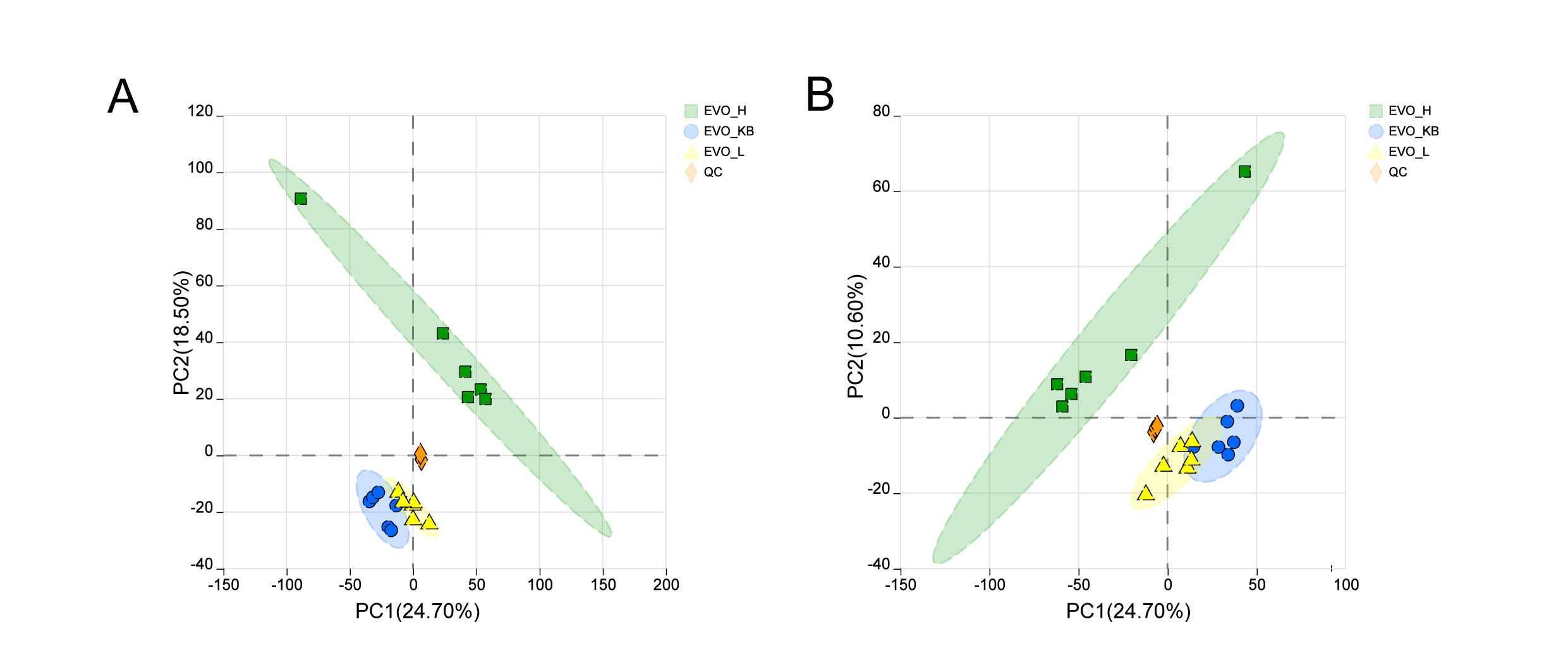


Supplementary Fig. 1

PCA score plots and permutation test results of metabolomic analysis on L02 cells. (A) ESI+ mode. (B) ESI- mode.


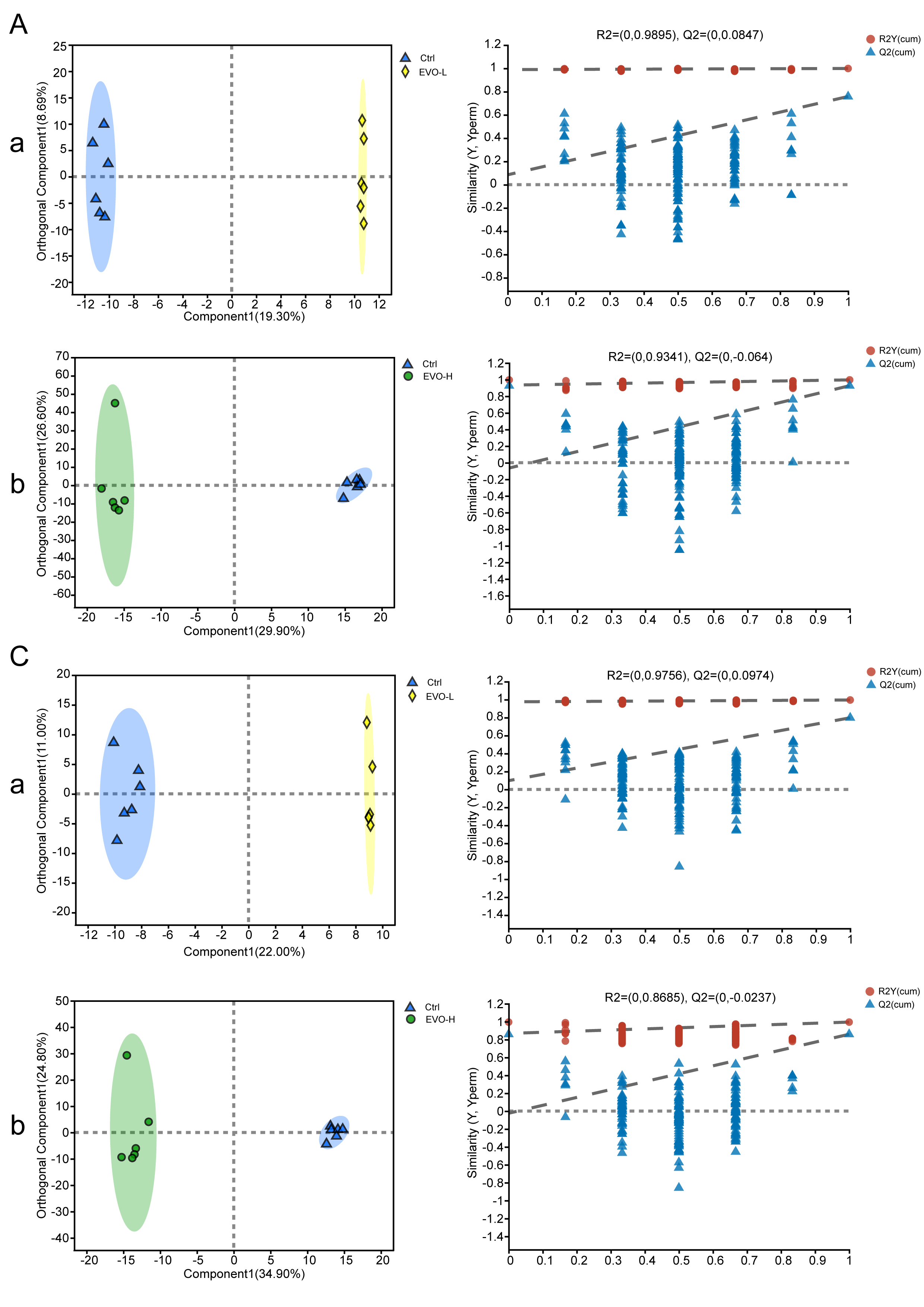


Supplementary Fig. 2

OPLS-DA score plots and permutation test results of metabolomic analysis on L02 cells. (A) ESI+ mode. (B) ESI- mode. a. Ctrl vs EVO-L. b. Ctrl vs EVO-H.


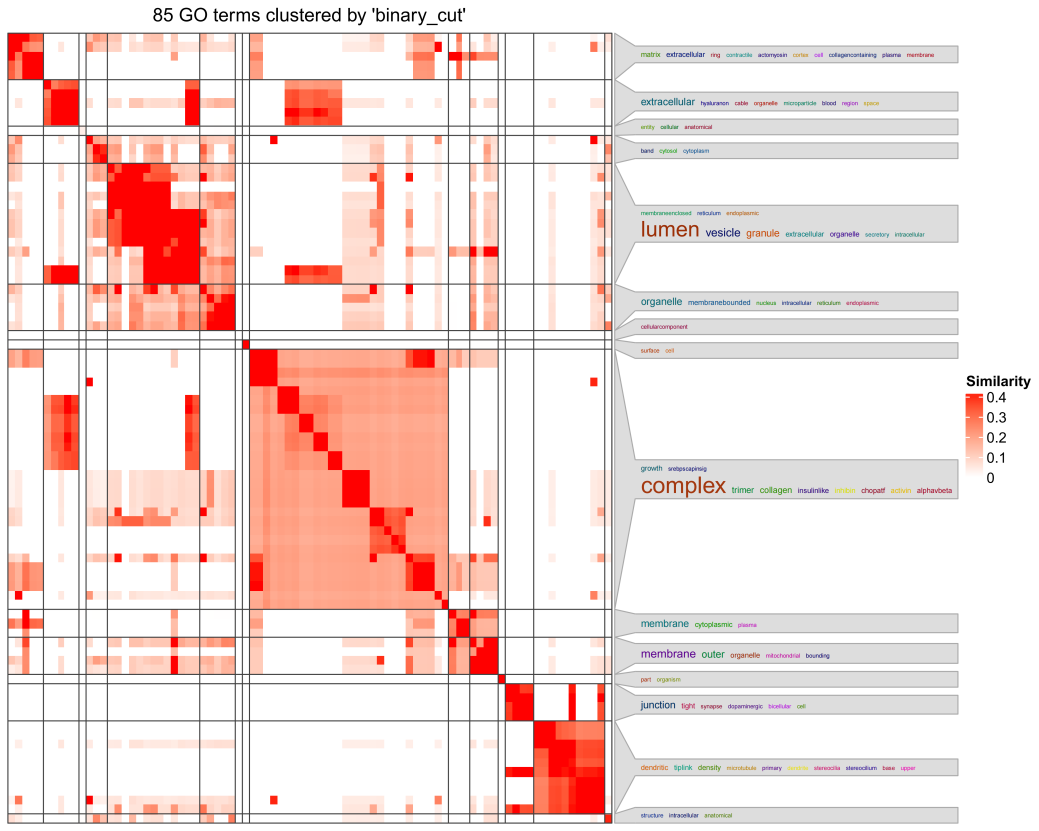


Supplementary Fig. 3

The items of Cellular Component (CC) category from GO annotation in transcriptomic analysis.


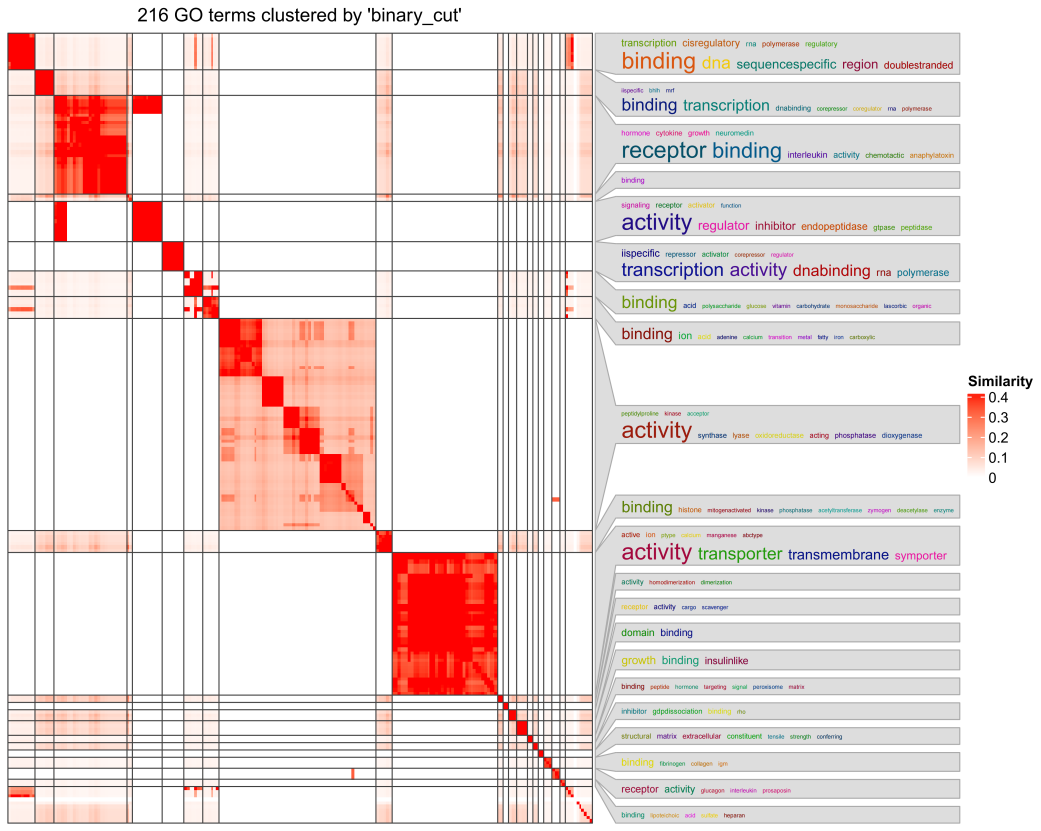


Supplementary Fig. 4

The items of Molecular Function (MF) category from GO annotation in transcriptomic analysis.

Supplementary Table 1

The primer sequences used in qPCR analysis.

| gene | Forward Primer sequence(5'-3') | Reverse Primer sequence(5'-3') |
| --- | --- | --- |
| BSEP | TGAATGGACTGTCGGTATCTGTG | CCACTGCTCCCAACGAATG |
| MRP2 | CTTGCTGTGGTCCAGTGTTTTAC | ATGGCGAATGGCAGACAA |
| CYP7A1 | AACAACCTGCCAGTACTAGATAGC | GTGTAGAGTGAAGTCCTCCTTAGC |
| CYP27A1 | GCCTCACCTATGGGATCTTCA | TCAAAGCCTGACGCAGATG |
| GAPDH | AGGTCGGTGTGAACGGATTTG | TGTAGACCATGTAGTTGAGGTCA |

Supplementary Table 2

Antibodies in Western blotting.

| Antibody | Manufacturer | Catalog Number |
| --- | --- | --- |
| β-actin | ABclonal | AC026 |
| PPARα | Santa | sc-398394 |
| p-p65 | Proteintech | 82335-1-RR |
| ZO-1 | Affinity | AF5145 |
| Occludin | abcam | ab216327 |
| Caspase3(human) | abcam | ab32351 |
| Caspase3(mouse) | Servicebio | GB11767C |
| BSEP | Proteintech | 67512-1-Ig |
| MRP2 | abcam | ab172630 |
| CYP7A1 | Proteintech | AF6657 |
| CYP27A1 | Beyotime | 14739-1-AP |
| TNFα | Servicebio | GB12188 |

Supplementary Table 3

The OPLS-DA results of metabolomic analysis on L02 cells.

| Scan mode | Comparison | R^2^X(cum) | R^2^Y(cum) | Q^2^ |
| --- | --- | --- | --- | --- |
| ESI+ | Ctrl vs EVO-L | 0.280 | 0.999 | 0.758 |
|  | Ctrl vs EVO-H | 0.565 | 0.997 | 0.927 |
| ESI- | Ctrl vs EVO-L | 0.329 | 0.996 | 0.798 |
|  | Ctrl vs EVO-H | 0.596 | 0.995 | 0.859 |
| Note: R^2^X(cum) = Cumulative explained fraction of X variation; R2Y(cum) = Cumulative explained fraction of Y variation; Q^2^ = Cumulative predicted fraction | | | | |
